# Core Mycobiome and Their Ecological Relevance in the Gut of Five *Ips* Bark Beetles (Coleoptera: Curculionidae: Scolytinae)

**DOI:** 10.3389/fmicb.2020.568853

**Published:** 2020-09-03

**Authors:** Amrita Chakraborty, Roman Modlinger, Muhammad Zubair Ashraf, Jiří Synek, Fredrik Schlyter, Amit Roy

**Affiliations:** ^1^EVA 4.0 Unit, Faculty of Forestry and Wood Sciences, Czech University of Life Sciences, Prague, Czechia; ^2^Excellent Team for Mitigation, Faculty of Forestry and Wood Sciences, Czech University of Life Sciences, Prague, Czechia; ^3^Department of Plant Protection Biology, Swedish University of Agricultural Sciences, Alnarp, Sweden

**Keywords:** OTUs, gut-mycobiont, fungal-biomarkers, core community, Scolytinae, symbiosis, bark beetles, *Ips*

## Abstract

Bark beetles are destructive forest pests considering their remarkable contribution to forest depletion. Their association with fungi is useful against the challenges of survival on the noxious and nutritionally limited substrate, i.e., conifer tissues. Fungal symbionts help the beetles in nutrient acquisition and detoxification of toxic tree secondary metabolites. Although gut is the prime location for food digestion and detoxification, limited information is available on gut-mycobiome of bark beetles. The present study screened the gut-mycobiont from six bark beetles (five *Ips* and one non-*Ips*) from Scolytinae subfamily using high-throughput sequencing and explored their putative role in symbiosis with the host insect. Results revealed the predominance of four fungal classes- Sordariomycetes, Saccharomycetes, Eurothiomycetes, and Dothidomycetes in all bark beetles. Apart from these, Agaricomycetes, Leothiomycetes, Incertae sedis Basidiomycota, Tremellomycetes, Lecanoromycetes, and Microbotryomycetes were also documented in different beetles. Five *Ips* bark beetles share a consortium of core fungal communities in their gut tissues consisting of 47 operational taxonomic units (OTUs) belonging to 19 fungal genera. The majority of these core fungal genera belong to the phylum Ascomycota. LEfSe analysis revealed a set of species-specific fungal biomarkers in bark beetles. The present study identified the gut mycobiont assemblage in bark beetles and their putative ecological relevance. An enriched understanding of bark beetle-fungal symbiosis is not only filling the existing knowledge gap in the field but may also unleash an unforeseen potential for future bark beetle management.

## Introduction

The evolutionary success of insects is based on myriad associations with microorganisms having complementary potential that is otherwise lacking in insects and restricts them when inhabiting an ecologically challenging niche or invading new environments (Linnakoski et al., 2012; Sun et al., 2013; Douglas, 2015; García-Fraile, 2018). Hence, insects serve as an excellent model system to study such evolutionary associations (i.e., symbiosis) concerning animal-plant ecology. Bark beetle (Coleoptera: Curculionidae: Scolytinae) and fungi association is one of such model systems. The evolution of bark beetle (BB)-fungi mutualisms is based on reciprocal interactions such as nutritional mutualisms, protective mutualisms, and dispersal mutualism (Biedermann and Vega, 2020). In BB exploiting sapwood (xylem), fungal associates often alleviate the deficiency of such plant tissue that is one of the most nutritionally-limiting and recalcitrant organic substrates on earth to thrive on (Filipiak and Weiner, 2014, 2017). The high abundance of substances like lignin, hemicellulose, cellulose (i.e., bio-polymers with rich carbon content) in the wood is not accessible to beetles until prior degradation of microbes, i.e., fungi (Kirk and Cowling, 1984).

Fungi often benefit coleopteran insects including BB by detoxifying plant allelochemicals (Dowd and Shen, 1990; Wang et al., 2013; Tsui et al., 2014; Zhao et al., 2019). It is believed that fungi and BB together involved in the exhaustion of host defense during the mass attack and subsequent tree-killing (Krokene and Solheim, 1996; Paine et al., 1997). Hammerbacher et al. (2013) showed blue-staining ascomycete *Ceratocystis polonica* helping the spruce BB, *Ips typographus* to break down the stilbene (phenolic compound) defenses of Norway spruce. The similar multipartite relationship was observed between symbiotic fungi (*Grosmannia clavigera*, *Leptographium longiclavatum*, *Ophiostoma montium*) and mountain pine beetle (*Dendroctonus ponderosae* Hopkins), where the presence of such fungal symbionts likely benefited the beetles under a wide range of environmental conditions (Ojeda Alayon et al., 2017). However, results from many other studies raise an argument against the importance of fungi for beetle fitness and killing the host trees as the pathogenic fungi are also found to be associated rather regularly with parasitic beetles that are less aggressive and do not kill the host conifers (Harrington, 1993; Wingfield, 1995). Furthermore, symbiotic fungus (i.e., *Candida nitratophila* isolated from *Ips typographus*) is also reported to oxidize pheromone compound *cis*-verbenol to *trans*-verbenol or verbenone. In return to all those favors, fungi benefit from dispersal by insect hosts (dispersal mutualism).

Bark beetles (BB) are economically important insects (Huang et al., 2020). The BB subfamily Scolytinae of weevils (Curculionidae) is composed of approximately 6000 species (Kirkendall et al., 2015). Association with fungi such as *Ophiostoma*, *Ceratocystiopsis*, *Ceratocystis*, and *Grosmannia* is very common among Scolytinae (Six, 2003; Kirisits, 2004; Zipfel et al., 2006). Some BB also associated with Basidiomycetes fungi (Whitney et al., 1987; Hsiau and Harrington, 2003). All strongly mutualistic associations are upheld generation after generation by vertically transmitting fungi through dedicated structures called mycangia (Six, 2020). Non-mycangial beetles transmit fungi via gut or body surface (Six, 2003; Stefanini, 2018). Ayres et al. (2000) showed that the N concentration of phloem tissues with *Dendroctonus frontalis* (mycangial beetle) larvae are twice than non-infested tissues indicating the importance of fungi for the growth of *D. frontalis* larvae. The same study also reveals that mycangial beetles (*D. frontalis*) construct smaller feeding galleries than non-mycangial beetle (*Ips grandicollis*), which is understandable considering the nutritional availability in host plant tissues influencing high or low consumption strategy for livelihood (Ayres et al., 2000).

Insect gut provides a typical environment for microbial inhabitation, including fungal colonization (Engel and Moran, 2013; Stefanini, 2018). Besides, herbivore insect gut is the site where plant allelochemical digestion, detoxification, and nutritional exchange occurs (Linser and Dinglasan, 2014). Gut-associated fungi can facilitate some or all of those vital processes inside the gut of its host (Dowd and Shen, 1990; Genta et al., 2006; DiGuistini et al., 2011; Wang et al., 2013; Itoh et al., 2018). Despite the functional importance of the gut inhabiting fungi in the adaptive physiology of its hosts, very few studies on BB have investigated the gut-associated fungal community exclusively. The whole-body microbiome studies, common in BB mycobiome research area, often represent a mix of biota from the digestive tract, external biota that escapes surface sterilization (mostly from mouthparts and entomogenous fungi from exoskeleton), and hemocoel and gonad rudiments (presence of fungi unknown).

Furthermore, basic information about the core fungal community in BB gut (subfamily: Scolytinae; *Ips* species) and their ecological role, including the metabolic interaction with the host is also limited. Hence, it is necessary to formulate studies on tissue-specific BB gut fungal assemblages, which are critical to a comprehensive understanding of the adaptive physiology of BB in general and their fungal symbionts inside the gut. For non-mycangial BB where gut serves as an essential site for carrying symbiotic microbes, such studies are even more crucial. Moreover, classic culture-dependent study methods are frequently biased to some specific groups of the fungal communities depending on the culture condition and laboratory processes involved downstream. Recent culture-independent, high-throughput next-generation sequencing-based screening of fungal communities provides an opportunity for exclusive identification and facilitate studying the ecological relevance of fungal species inhabiting inside BB gut that are never detected before. Furthermore, the gut fungal community assemblages may differ in same beetle species due to geographical and climatic differences between sampling sites, other biotic and abiotic factors including tree tissue, season or time of sampling, methodological strategies, and even cross-contamination during molecular processing or sample handling as described clearly in the review by Linnakoski et al. (2012). Hence, more studies are needed in different geographic locations with the same or different BB species to obtain a quick, comprehensive overview of such multipartite BB-fungal associations, which may expose surprising opportunities in bark beetle management (Popa et al., 2012).

Taken together, the current understanding of BB-fungal association and the ecological relevance of the gut tissue-specific association is incomplete, although relevant from both eco-evolutionary and applied perspectives. In Czech Republic, *Ips typographus* (IT), *I. duplicatus* (ID), and *Polygraphus poligraphus* (PP) are common spruce feeding beetles. *Ips typographus* is one of the most damaging pests in Europe, causing landscape-level mortality of spruce (Lieutier et al., 2004). *I. duplicatus* attacks green standing trees only whereas *Polygraphus poligraphus* (PP, non-*Ips*) likes spruce trees growing under dense and shaded condition (Kolk et al., 1996). Among the pine feeding beetles in Czech Republic, *Ips acuminatus* (IAC) causes considerable damage on the top and branches of Scots pine (Davydenko et al., 2017). Being a secondary pest of pine, *Ips sexdentatus* (SX) attacks stressed and weekend tress (Kolk et al., 1996). *Ips cembrae* (IC), secondary pest of European larix population, prefers wind-blown and dying trees for colonization. However, during an outbreak, IC can colonize not only on larch but also on spruce (Kolk et al., 1996). All these beetles can attack green standing trees under drought conditions and thus possess an increasing threat to forests. Hence, in the present study, we attempt to study gut tissue-specific fungal community assemblages in six economically important bark beetles (BB) from Scolytinae subfamily [IT, ID, IC, SX, IAC, and PP] collected from forests in Czech Republic using culture-independent molecular ecological approaches based on internal transcribed spacer (ITS) sequencing. Subsequently, for the core fungal communities inside the gut irrespective of beetle species, sampling location or host plant are identified, and their potential function within the gut communities is explored. Such information fills the existing knowledge gap and provides a sound scientific basis for a future shotgun or meta-transcriptomics studies followed by downstream functional studies to unravel the fine-tuning of the metabolic exchange between gut inhabiting mycobionts and their insect host.

## Materials and Methods

### Bark Beetle Collection, Dissection, and Gut DNA Extraction

**FIGURE 1:**
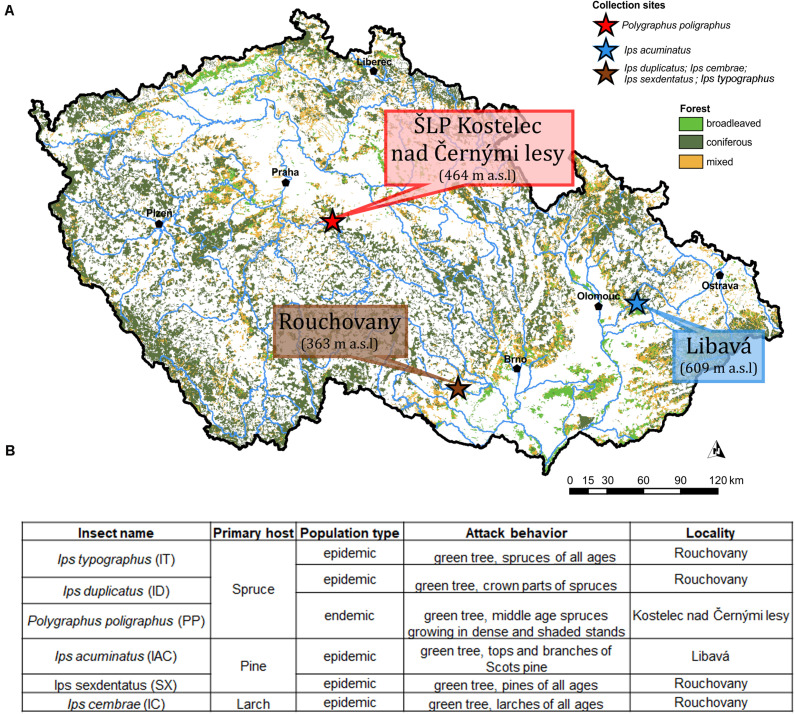
**(A)** Sampling site. The different sampling location for bark beetle collection. *Ips duplicatus* (ID), *Ips typographus* (IT), *Ips sexdentatus* (SX), and *Ips cembrae* (IC) are collected from Rochovany, Czech Republic. *Polygraphus poligraphus* (PP) is collected from Kostelec nad Černými lesy, and *Ips acuminatus* (IAC) is collected from Libavá, Czech Republic. **(B)** Ecology, population phase and attack behavior of the collected beetles (Lieutier et al., 2004).

Emerged adult bark beetle (Coleoptera: Curculionidae: Scolytinae) were sampled from infested trees during May and June 2018 from local forests in the Czech Republic. Precisely, *Ips typographus* (L.) (IT), *Ips cembrae* (IC), *Ips sexdentatus* (SX), *Ips duplicatus* (ID), were collected from Rouchovany (49°04′08.0″N 16°06′15.4″E, under State Forest Enterprise); *Polygraphus poligraphus* (PP) was collected from Kostelec nad Černými lesy Kostelec nad Èernımi lesy (50°00′07.2″N 14°50′56.3″E, under Military Forest Enterprise) and *Ips acuminatus* (IAC) was collected from Libavá (49°40′18.8″N 17°31′44.1″E, under School Forest Enterprise) in the Czech Republic (Figure 1A). The ecology and population phase of the collected beetles were summarized in Figure 1B. More than 120 living and healthy beetles were collected and pooled from logs belonging to more than eight infested trees per locality to make six biological replicates. Bark beetle species identification was performed based on morphology and published classical taxonomic work (Pfeffer, 1955; Nunberg, 1981). Beetles were stored at 4°C until shock frozen under liquid nitrogen for future use. Due to the pooling of beetles during sampling, the individual colony specific variability of the beetle gut fungal diversity could not be explored. Alternatively, such an approach somewhat reduces the stochastic variations connected with the heterologous natural tissue material (i.e., bark beetles from different colonies) and enables the separation of transient OTUs using statistical methods.

Randomly selected bark beetles were surface sterilized following the standard protocol and dissected under sterile conditions in the biosafety cabinet under the microscope with a sterile entomological dissection kit. Beetle gut with apparent nematode infection was discarded. Beetle gut tissues (10 guts per replicate) were homogenized, and microbial DNA was extracted using PureLink Microbiome DNA Purification Kit from Invitrogen company. Beetle gut DNA was quantified using Qubit 2.0 Fluorometer (Thermo Scientific), and the integrity was assessed by 1% agarose gel electrophoresis. High-quality DNA from all beetle species (six biological replicates per species) was sent for high-throughput amplicon sequencing (Internal transcribed spacer, ITS) at Novogene Company, China.

### Internal Transcribed Spacer (ITS) Sequencing

Amplicon sequencing was done at the Novogene company, China, following the standard company protocol. Precisely, after diluting the DNA to 1 ng/μL in sterile water, fungal ITS genes of distinct regions (ITS2) were amplified using a specific set of primers (ITS3, ITS4; amplicon size 386 bp) (White et al., 1990) with unique barcodes. PCR reactions were performed using Phusion High-Fidelity PCR Master Mix (New England Biolabs). PCR products with amplification between 400 and 450 bp were selected and mixed in equidensity ratios for gel purification. No template control in PCR reaction did not show any amplification; hence it was not used for gel purification and further library preparation. After purification of PCR products using the Qiagen Gel Extraction Kit (Qiagen, Germany), sequencing libraries were created using NEBNext Ultra DNA Library Pre-Kit from Illumina, and index codes were ligated. The library quantity and quality were analyzed using Qubit 2.0 Fluorometer (Thermo Fisher Scientific), Q-PCR and in Agilent Bioanalyser 2100 system respectively. The libraries were sequenced to generate 250 bp paired-end reads using an Illumina platform.

### ITS Data Analysis

#### Paired-End Reads Assembly and Quality Control (QC)

After read assignment to different samples, barcode, and primer sequence removal, paired-end reads were assembled using FLASH (V1.2.7)^1^ (Magoč and Salzberg, 2011). Quality filtering was performed to obtain high-quality clean tags applying pre-set parameters (Bokulich et al., 2013) in QIIME (V1.7.0)^2^ (Caporaso et al., 2010). UCHIME algorithm^3^ (Edgar et al., 2011) was used to detect chimera sequences^4^ taking the UNITE database (Nilsson et al., 2019) as a reference, and subsequently, the chimera sequences were removed (Haas et al., 2011), and the effective Tags were finally collected.

#### OTU Clustering and Annotation

Data analysis was conducted using UPARSE software (UPARSE v7.0.1001)^5^ (Edgar, 2013). After assigning all sequences with ≥ 97% similarity to the same OTU, obtained OTUs were blasted against the UNITE Database for fungal species annotation. Multiple sequence alignment (MSA) was performed to explore the phylogenetic relationship of different OTUs using MUSCLE software (Version 3.8.31)^6^ (Edgar, 2004). During the analysis, the singletons (*n* < 2) were discarded, and OTU abundance was normalized using the sequence number corresponding to the sample with the least sequences. The alpha and beta diversity analysis was subsequently performed based on the normalized OTU abundance data.

#### Alpha Diversity

The complexity of fungal species diversity (Alpha diversity) was estimated for each sample using standard indices such as observed-species, sequence depth (Good’s coverage) (Chao et al., 1988), community richness (Chao1, ACE), diversity (Shannon, Simpson) (Magurran, 1988). These indices in all 36 tested samples were calculated in QIIME (Version 1.7.0) (Caporaso et al., 2010) and presented using R software (Version 2.15.3; R Core Team, 2013, Vienna, Austria) (R Core Team, 2013).

#### Beta Diversity

The differences in fungal species complexity in different beetle samples (Beta diversity) (Lozupone et al., 2007) was estimated using QIIME software (Caporaso et al., 2010) (Version 1.7.0). Weighted and unweighted UniFrac distance matrices were used to measure the dissimilarity coefficient between pairwise samples. Non-metric multidimensional scaling analysis (NMDS) (Oksanen et al., 2010) was performed to get the principal coordinates and visualize the complex, multidimensional data. Unweighted Pair-group Method with Arithmetic Means (UPGMA) Clustering (Knight et al., 2010) was done to interpret the distance matrix using average linkage in QIIME software (Version 1.7.0) (Caporaso et al., 2010). Variation analysis of fungal community structure between different bark beetle (BB) gut was assessed by standard statistical methodologies such as Analysis of Similarity (ANOSIM) (Clarke, 1993), Multi-response permutation procedure (MRPP) analysis (Cai, 2006) and ADONIS (Anderson, 2001) using R software (Vegan package) (Oksanen et al., 2007). Furthermore, Metastats (Paulson et al., 2011) was used to observe the variation in fungal species between the different BB sample groups. The significance of observed fungal species abundance differences among groups was further evaluated by *p*-value calculated by the method of permutation test and *q*-value calculated by the method of Benjamini and Hochberg False Discovery Rate (FDR) (White et al., 2009). Lastly, LEfSe [linear discriminant analysis (LDA) Effect Size] analysis was performed to detect key fungal species with a significant intra-group variation among beetle sample groups using LEfSe software (Segata et al., 2011).

## Results

### Sequencing Statistics

Illumina paired-end sequencing of the gut tissue of six bark beetles from the Scolytinae subfamily yielded a total of 4,564,713 reads, of which 4,400,177 reads were obtained after quality control tests (sequences with Phred Quality score < 30 were discarded). The fungal ITS2 clean-reads observed per bark beetle species were used for further downstream bioinformatic data processing (Supplementary Excels 1, 2).

### Gut Fungal Diversity

#### OTU Abundance

**FIGURE 2:**
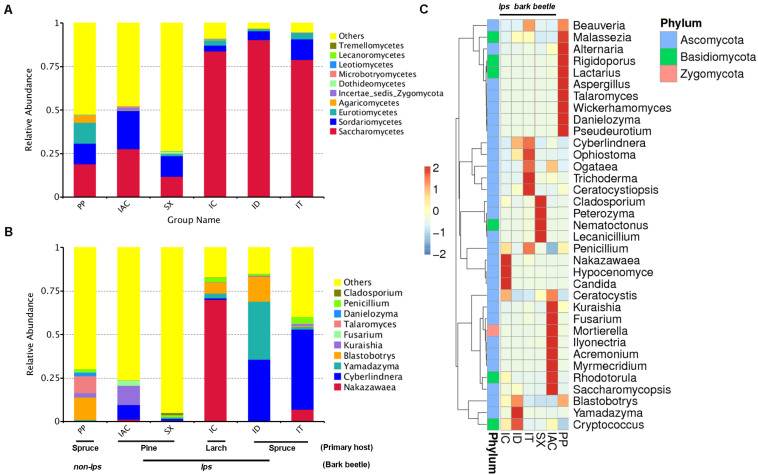
Fungal diversity observed in the gut of different bark beetles. **(A)** The bar plot illustrating the relative abundance of the gut mycobiome at the class level (top 10). “Others” signifies the total relative abundance of the rest of the class. **(B)** The histogram depicting the relative abundance of top 10 fungal genera in the bark beetle gut. Likewise, “Others” represents the total relative abundance of rest of the fungal communities in the beetle gut. **(C)** Heatmap displaying the abundance of 35 dominant fungal genera in the gut of the bark beetles. The color gradient shows the relative OTU abundance for each bark beetle where the darker color signifies higher abundance and lighter color indicates a low abundance of the particular fungal genera.

The comprehensiveness of sampling was represented by the Good’s coverage estimator (>99%), and the rarefaction curves that tend to attain a plateau indicating most of the gut fungal diversity in the bark beetles (BB) were sequenced (Supplementary Table 1 and Supplementary Figure 1). The number of observed species was significantly higher in PP (176.5 ± 6.84) compared to all other beetles (*P* < 0.01) (Supplementary Table 1). A total of 955 fungal OTU (operational taxonomic unit) clusters at 97% similarity were observed in the six different bark beetles (Supplementary Excel 3). The gut mycobiome represented using GraPhlAn revealed the predominance of four different classes- Sordariomycetes, Saccharomycetes, Eurothiomycetes, and Dothidomycetes that were present in all BB (Supplementary Figures 2,3). Apart from these classes, Agaricomycetes, Leothiomycetes, Incertae sedis Basidiomycota, Tremellomycetes, Lecanoromycetes, Microbotryomycetes were also observed in different BB (Supplementary Figures 2,3). However, the GraPhlAn display of IAC was represented at the phylum level due to the presence of an increased number of different classes compare to other beetles (Supplementary Figure 3). Considering the spruce feeding beetles, Saccharomycetes was most abundant in ID (90.3%) followed by IT (78.9%) and lowest in PP (19%) whereas Sordariomycetes showed similar abundance in PP (11.6%) and IT (11.8%) compare to ID (5.1%). Furthermore, PP showed a high relative abundance of Eurothiomycetes (12.1%) and Agaricomycetes (4.2%) compared to other BB (Figure 2A and Supplementary Excel 4). Nonetheless, comparing the pine feeding beetles, IAC showed a similar relative abundance of Saccharomycetes (27.7%) and Sordariomycetes (21.8%) in the gut. Likewise, SX also followed a similar trend (Saccharomycetes- 11.8% and Sordariomycetes- 11.7%) (Figure 2A and Supplementary Excel 4). Additionally, the larch feeding IC documented the high abundance of Saccharomycetes (83.8%) followed by Sordariomycetes (3.4%) and Eurothiomycetes (2.8%) (Figure 2A and Supplementary Excel 4).

The evolutionary tree illustrated the relative abundance of top 100 fungal genera, and further, the top 20 fungal species identified in our study were represented in the taxonomic tree (Supplementary Figure 4). The relative abundance of Ascomycota was highest, followed by Basidiomycota and Zygomycota (Supplementary Excel 4). In particular, the presence of *Cladosporium, Penicillium, Danielozyma, Talaromyces, Fusarium Kuraishia, Blastobotrys, Yamadazyma, Cyberlindnera*, and *Nakazawaea* belonging to Ascomycota phylum were among the most abundant fungal genera present in the gut of BB (Figure 2B and Supplementary Excel 4). Other dominant genera include *Beauveria, Malassezia, Alternaria, Aspergillus, Wickerhamomyces, Ophiostoma, Ogataea, Trichoderma, Ceratocystiopsis, Peterozyma, Lecanicillium, Candida, Ceratocystis, Mortierella, Rhodotorula, Saccharomycopsis*, and *Cryptococcus* (Figure 2C and Supplementary Excel 4). Furthermore, the ternary plots representing the predominant fungal genera (top 10) among the spruce feeding BB revealed the predominance of *Yamadazyma* in ID (33.3%). In contrast, *Nakazawaea* was abundant in IT (6.9%) and *Talaromyces* in PP (9.6%). Additionally, *Cyberlindnera* was documented to be prevalent in both IT (46%) and ID (35.1%) whereas, *Blastobotrys* was dominant in ID (14.4%) and PP (12.8%) (Supplementary Figure 5 and Supplementary Excel 4). Furthermore, considering the pine and larch feeding beetles (IAC, SX, and IC), IC showed the predominance of *Nakazawaea* (70%), *Blastrobotrys* (6.2%), *Yamadazyma* (2.7%) and *Penicillium* (2.4%). Nevertheless, *Cyberlindnera* (8.4%), *Kuraishia* (10.9%), and *Fusarium* (3.17%) were most abundant in IAC whereas, *Cladosporium* was highly present in SX (1.2%) (Supplementary Figure 5 and Supplementary Excel 4).

#### Alpha Diversity

**FIGURE 3:**
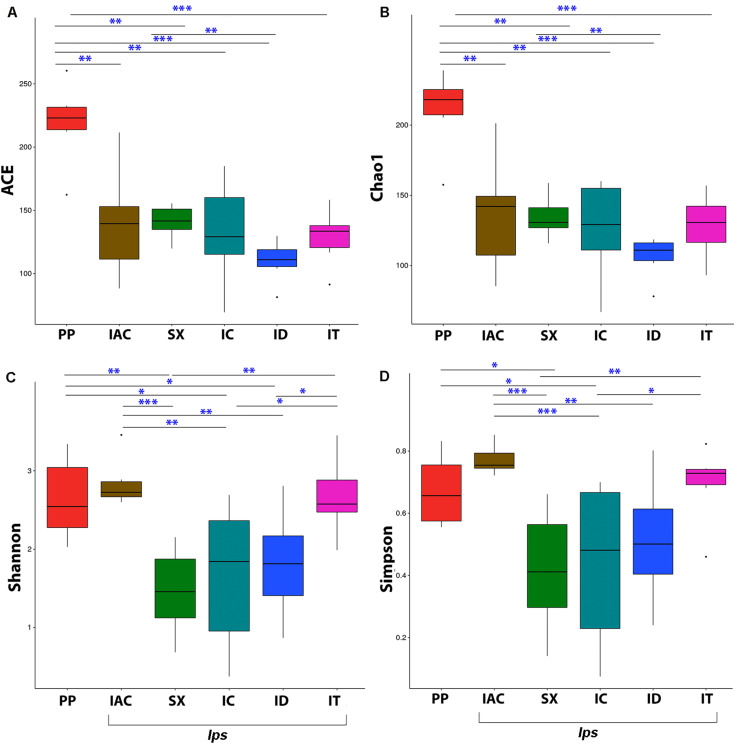
Alpha diversity indices representing the fungal community richness and diversity among the six different bark beetles. The expected richness estimated by **(A)** ACE analysis and **(B)** Chao1 analysis indicated significant variation between *Polygraphus poligraphus* (PP) and other spruce feeding beetles while no such difference in community richness was observed between pine feeding beetles (IAC and SX). The gut fungal diversity illustrated by **(C)** Shannon index and **(D)** Simpson index documented similar diversity measures between *Polygraphus poligraphus* (PP) and *Ips typographus* (IT) compared to *Ips duplicatus* (ID) whereas *Ips acuminatus* (IAC) represented higher diversity than *Ips sexdentatus* (SX). The statistical analysis for the significant differences between groups is done by Wilcoxon signed-rank test where “^∗^” denotes *p* < 0.05, “^∗∗^” designates *p* < 0.01 and “^∗∗∗^” resembles *p* < 0.001.

The fungal community richness and diversity present within the gut tissue of the BB was indicated by the alpha diversity indices (Figure 3 and Supplementary Table 1). The Shannon and Simpson indices representing the community diversity in the gut tissue of spruce feeding BB revealed similar diversity in between *Ips typographus* (IT) (Shannon-2.67 ± 0.20 and Simpson-0.69 ± 0.05) and non-*Ips* BB, *Polygraphus poligraphus* (PP), (Shannon-2.64 ± 0.21 and Simpson- 0.67 ± 0.05) compared to *Ips duplicatus* (ID) (Shannon- 1.81 ± 0.28 and Simpson- 0.51 ± 0.08) (Figures 3C,D). Interestingly, the expected fungal richness (Chao1 and ACE; Wilcoxon signed-rank test) among these spruce feeding beetles documented higher richness in PP (Chao1- 210.72 ± 11.63 and ACE- 218.96 ± 13.20) compared to IT (Chao1- 128.19 ± 9.24 and ACE-128.75 ± 9.24) and ID (Chao1- 109.76 ± 6.76 and ACE- 109.76 ± 6.76) (Figures 3A,B). Furthermore, considering the pine feeders, *Ips acuminatus* (IAC) (Shannon- 2.84 ± 0.13 and Simpson- 0.77 ± 0.02) showed significantly higher gut fungal diversity than *Ips sexdentatus* (SX) (Shannon-1.46 ± 0.23 and Simpson-0.42 ± 0.08) (Figures 3C,D). However, no significant difference in fungal richness was documented between IAC (Chao1- 136.38 ± 16.99 and ACE- 139.74 ± 17.68) and SX (Chao1- 134.34 ± 6.15 and ACE- 140.90 ± 5.45) (Figures 3A,B). Additionally, the gut mycobionts of *Ips* beetles showed similar community richness and diversity among them compared to PP (Figure 3 and Supplementary Table 1).

**FIGURE 4:**
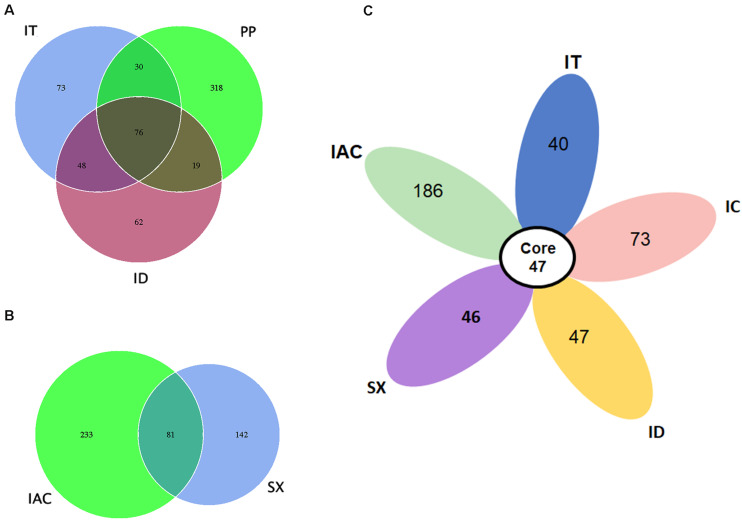
Core gut mycobiome. **(A)** Venn diagram showing the gut fungal OTU distribution between the spruce feeding bark beetles (IT, PP, and ID) where the number of OTUs (common or unique) shared among the beetles is denoted. **(B)** Venn diagram displaying the number of common and unique OTUs between the pine feeding beetles (IAC and SX). **(C)** Flower diagram showing the presence of 47 core OTUs shared among the five different *Ips* bark beetles.

Our data suggest that the BB share a consortium of core fungal communities in their gut tissues (Figure 4). The core mycobionts present in the spruce feeding BB (PP, IT, and ID) consists of 76 OTUs belonging to 22 fungal genera (Figure 4A and Supplementary Excel 5). Similarly, 81 OTUs assigned to 25 genera were shared between the pine beetles (SX and IAC) (Figure 4B and Supplementary excel 6). Interestingly, comparing the five *Ips* bark beetles the occurrence of 47 core fungal OTUs were observed that were assigned to 19 fungal genera constituting *Cyberlindnera, Rhodotorula, Saccharomycopsis, Yamadazyma, Blastobotrys, Talaomyces, Fusarium, Kuraishia, Penicillium, Cladosporium, Candida, Ogataea, Cryptococcus, Ceratocystis, Ophiostoma, Trichoderma, Malassezia, Cuniculitrema, and Beauveria* (Figure 4C and Supplementary excel 7). Nevertheless, the core fungal consortium compared among all of the six BB (PP, IT, ID, IC, IAC, and SX) revealed the presence of 37 common OTUs predominantly belonging to 16 fungal genera (Supplementary Figure 6 and Supplementary Excel 8). Besides, the core mycobiome present in all the beetles, the occurrence of other fungal genera were also documented in this study. The existence of such fungal communities might be acquired from the environment as fungal spores or during feeding. Among these fungal communities, *Peterozyma* and *Hypocenomyce* were observed in the BB from Rouchavany (SX, IC, IT, and ID), whereas *Wickerhamomyces, Myrmecridium*, and *Lactarius* were documented in PP (Kostelec) and IAC (Libavá). Furthermore, *Grapium* was found in all the spruce feeding BB (PP, IT, and ID), and *IIyonectria* and *Coprinellus* were observed in pine (IAC, SX) and larch feeding (IC) bark beetles, respectively (Supplementary Excel 4).

#### Beta Diversity

**FIGURE 5:**
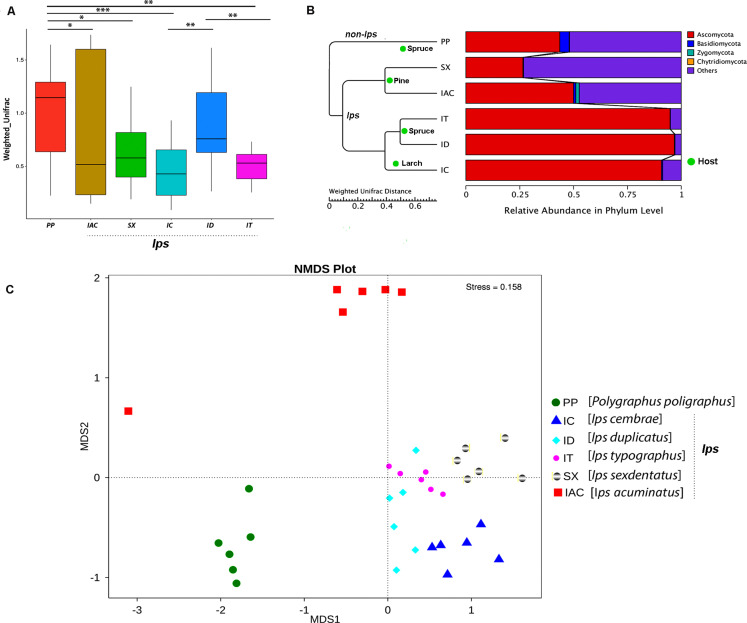
Beta diversity analysis. **(A)** The boxplot representing the diversity between the bark beetles based on the Weighted Unifrac distances showing significant differences between the PP and other bark beetles. The statistical analysis for significance is performed by Wilcoxon signed-rank test where the significance level at *p* < 0.05 is denoted by “^∗^,” “^∗∗^” designates *p* < 0.01 and “^∗∗∗^” signifies *p* < 0.001. **(B)** The WPGMA (Weighted pair group method with arithmetic mean) tree cluster based on Weighted Unifrac distance illustrates the similarity in gut fungal communities among the *Ips* species of bark beetles (IT, ID, IC, SX, and IAC) compare to non- *Ips* bark beetle PP. Additionally, bark beetles feeding on similar conifer host are grouped. The WPGMA tree displayed with the relative abundance of the gut mycobiome at phylum level. **(C)** Non-metric multidimensional scaling analysis (NMDS) representing the variation in the gut fungal communities between the six bark beetles. The data points in the same color denote the same bark beetle species. The different bark beetles are denoted by different symbols. The bark beetles collected from Rouchovany (IT, ID, IC, SX) are clustered together and are significantly different from the bark beetles sampled from other locations, Kostelec (PP) and Libavá (IAC).

The beta diversity calculated based on Weighted and Unweighted UniFrac distances reflects the differences in the gut mycobiome of six different BB (Figure 5 and Supplementary Figure 7). The box plot based on the Weighted UniFrac distances between the samples suggested that among the spruce feeding BB, the gut fungal communities in IT were significantly different from ID and PP (*p* < 0.01) (Figure 5A). However, no significant variation was observed between ID and PP. Furthermore, considering the larch feeding beetle, IC, showed a substantial difference in the gut mycobionts compare to ID (*p* < 0.01) and PP (*p* < 0.001). In contrast, no significant differences were documented between the pine feeding beetles (IAC and SX) (Figure 5A). However, there was considerable variation between PP and pine feeding BB (IAC, SX) (p < 0.05). Interestingly, the hierarchical clustering based on the Weighted Unifrac distances clustered all the *Ips* species together in one clade and placed PP (non- *Ips* species) in a different clade (Figure 5B) demonstrating the convergence of fungal association in *Ips* species. Furthermore, the influence of host tree feeding on the BB gut mycobiome was also observed where pine feeding (IAC and SX), spruce feeding (IT and ID), and larch feeding (IC) *Ips* beetles clusters together within the same clade (Figure 5B).

Alternatively, Non-Metric Multidimensional Scaling (NMDS) analysis revealed the environmental influence on the beetle gut communities resulting from different sampling locations. The NMDS plot illustrated differences in the gut mycobionts of bark beetles (BB) from Rouchavany (IT, ID, IC, and SX), Kostelec (PP), and Libavá (IAC) representing three clusters (Figure 5C). Such observation explaining the influence of the site-specific environmental conditions on the gut communities need further experimental validation.

**FIGURE 6:**
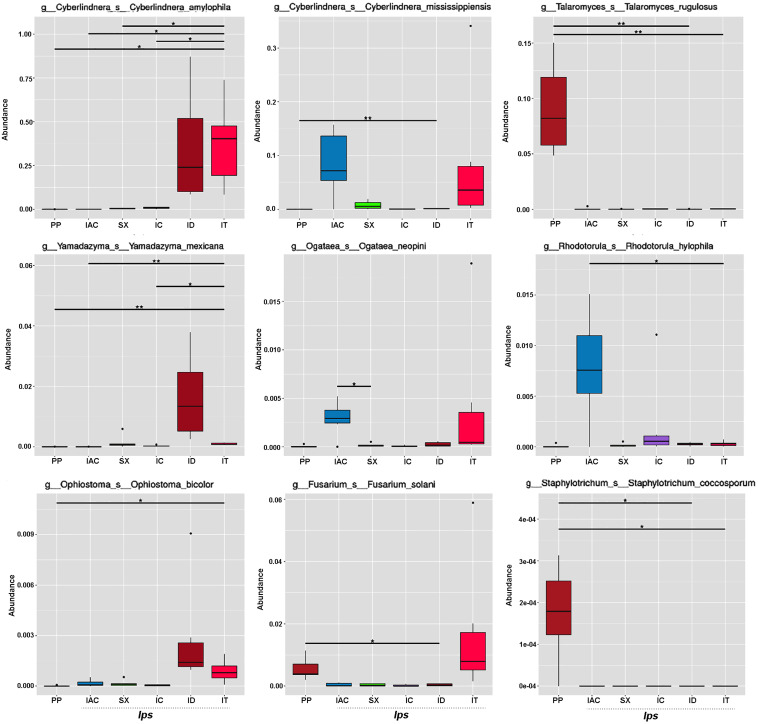
Metastats analysis. The barplot represents the fungal species-specific variation (top 9 species) in the gut of the bark beetles. The significant differences in species abundance among beetles are evaluated by the FDR test. The horizontal line designates the two groups with significant variation. “*” represents significant difference at *q* < 0.05 while “**” denotes significance at *q* < 0.01.

The fungal species-specific variation present in the gut of bark beetles represented by Metastat analysis revealed the preponderance of *Cyberlindera amylophila, Yamadazyma mexicana*, and *Ophiostoma bicolor* in the spruce feeding beetles from Rouchavany (IT and ID). In contrast, *Talaromycetes rugulosus*, *Staphylotrichum coccosporum* were significantly abundant in PP (Kostelec site) while, *Fusarium solani* was prevalent in both PP and IT. Furthermore, *Ogateae neopine, Rhodotorula hylophila*, and *Cyberlindera mississippiensis* were highly abundant in IAC (Figure 6). Statistical analysis (ANOSIM, MRPP, and ADONIS) illustrates significant variation between the fungal communities’ present in the six BB under study (Supplementary Tables 2,3). The gut mycobionts differed considerably between BB with different conifer feeding and were also influenced by the environment due to different collection site. Nevertheless, our data showed that the fungal communities associated with the BB were unique among different beetle species. Although the NMDS analysis documented the clustering of the bark beetles collected from the same site nearby, there were also noteworthy differences in the gut mycobiome between these beetles (Figure 5C and Supplementary Tables 2,3).

**FIGURE 7:**
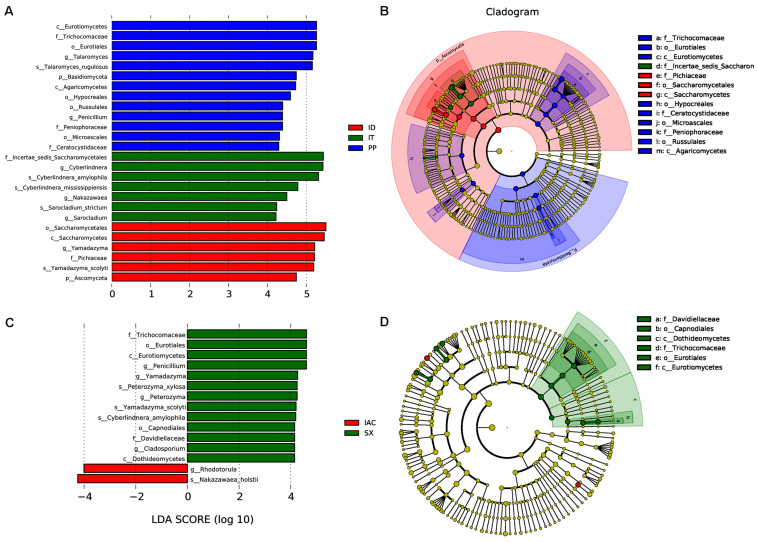
LEfSe analysis. **(A)** The histogram of the Linear discriminant analysis (LDA) scores illustrating the differentially abundant fungal communities in the spruce feeding bark beetles (IT, ID, and PP). The LDA score at log 10 > 4 is set as threshold and the length of each bin, i.e., LDA score represents the extent to which the fungal biomarker differs among the groups. **(B)** The cladogram illustrating the presence of fungal biomarkers that are significantly different between the spruce beetles. **(C)** The LDA score plot (log 10 > 4 as the threshold) representing the significantly abundant gut fungal communities in the pine feeding beetles (IAC and SX). **(D)** The cladogram describing the fungal biomarkers in pine beetles. The circles representing from inside to outside denotes different taxonomic level (phylum to genus). Each circle resembles a distinct taxon at the respective taxonomic level. The relative abundance of a particular taxon is proportional to the size of that circle. The color of the fungal biomarkers is according to the color of corresponding bark beetle while yellowish-green dots designate the non-significant fungal species. Red and green nodes denote that these fungi contribute highly to the group. Letters above the circles denote the fungal biomarker.

Moreover, the presence of significantly abundant fungal communities evaluated by Linear discriminant analysis effect size (LefSe) was described as important fungal biomarkers. The LDA (Linear discriminant analysis) scores [(log10) > 4 as threshold] represented by the histogram were used for the estimation of the biomarkers (Figure 7). The LDA score reflected the significant abundance of fungal classes that include- Eurotiomycetes, Agaricomycetes, Saccharomycetes along with fungal families belonging to Trichocomaceae, Peniophoraceae, Ceratocystidaceae, and Pichiaceae in the spruce feeding BB (IT, ID, and PP) (Figure 7A). Furthermore, the prevalence of fungal species belonging to Pichiaceae, Saccharomycetales, and Sccharomycetes was identified as biomarkers for ID. In contrast, Incertae sedis Saccharomycetales were abundant in IT. Additionally, the fungal communities belonging to Trichocomaceae, Eurotiomycetes, Hypocreales, Ceratocystidaceae, Microascales, Peniophoraceae, Russulales, and Agaricomycetes were observed as dominant markers in PP (Figure 7B). While in the pine feeding BB (IAC and SX), the histogram of the LDA scores illustrated the significant abundance of the fungal class belonging to Eurotiomycetes and Dothideomycetes and the families Trichocomaceae and Davidiellaceae in SX whereas fungal genera *Rhodotorula* and *Nakazawaea* in IAC (Figure 7C). Moreover, the cladogram indicated Davidiellaceae, Capnodiales, Dothideomycetes, Trichocomaceae, Eurotiales, and Eurotiomycetes as biomarkers in pine feeding beetle, SX (Figure 7D).

Taken together, the histogram representing all the six bark beetles (Supplementary Figure 8), illustrated the significant abundance of *Penicillium* and *Peterozyma* in SX while Microbotryomycetes, Sordariomycetes, Saccharomycetaceae, Ophiostomataceae, Nectriaceae, Plectosphaerellaceae, *Fusarium, Rhodotorula, Nakazawaea*, Incertae sedis Sordariomycetes in IAC. The fungal communities belonging to Eurotiomycetes, Agaricomycetes, Trichocomaceae, Trichomonascaceae, Ceratocystidaceae, Peniophoraceae were highly present in PP. Moreover, IT documented the significant abundance of Incertae sedis Saccharomycetales and *Cyberlindera*, whereas *Yamadazyma*, Microascaceae, Saccharomycetales were observed ID. The larch feeding BB (IC) showed the presence of Pichiaceae, *Candida*, and *Nakazawaea* in the histogram of LDA scores (Supplementary Figure 8A). Consequently, the overall cladogram representing the biomarkers from all the six bark beetles documented Incertae sedis Sordariomycetes, Ophiostomataceae, Plectosphaerellaceae, and Nectriaceae as markers in IAC while Pichiaceae in IC. The fungal order Saccharomycetales and family Microascaceae were the biomarkers in ID, whereas Incertae sedis Saccharomycetales was the dominant marker for IT. The non- *Ips* BB, *Polygraghus poligraphus* (PP) demonstrated Trichocomaceae, Trichomonascaceae, Ceratocystidaceae, Peniophoraceae as biomarkers (Supplementary Figure 8B).

## Discussion

Fungi are often associated with bark beetles (BB) as their symbionts where fungi are either carried in specialized mycangia or on their exoskeleton (Klepzig and Six, 2004; Six, 2012). Most of the previous BB-fungal symbiosis studies are conducted non-tissue specific manner, i.e., taking the whole bark beetles comprising endo and exo-mycobiome (Kirisits, 2007; Six, 2012). Hence, we have a limited understanding of the tissue-specific assemblage and interaction between BB and its fungal associates, which is an important aspect for having a better understanding of symbiosis. Not surprisingly, BB gut serves as a rich source of mycobionts assemblage. As insect gut is the prime site for digestion and detoxification of food (Linser and Dinglasan, 2014), often gut-associated mycobionts play a crucial role in those processes and decide the fate of host-derived compounds (Itoh et al., 2018). Hence, the present study is designed to screen the gut fungal diversity in five *Ips* and one non-*Ips* bark beetles (BB) collected from three different sites (Figure 1) in Czech Republic. Our data reported higher fungal diversity in the gut of *Ips acuminatus* (IAC) and *Ips typographus* (IT) compared to other tested *Ips* bark beetles and *Polygraphus poligraphus* (PP) (Supplementary Table 1). The pine feeding BB, *Ips acuminatus* (IAC) documented higher gut fungal diversity may be due to their fungus feeding behavior at the larval stage. However, there is no apparent difference in the gut mycobiome richness compare to other pine inhabitants, *Ips sexdentatus* (SX) (Figure 3). Congruently, *Ips acuminatus* (IAC) considered as less aggressive yet attacked healthier coniferous trees (Wermelinger et al., 2008) compare to the other secondary pest on pine, *Ips sexdentatus* (SX) (Pineau et al., 2017). Hence, the higher mycobiome diversity in IAC could thereby contribute to coping with the toxic defensive pine compounds (Davis et al., 2018) and nutritional acquisition during development (Villari et al., 2012). On the contrary, the aggressive secondary BB (Six, 2020), *Ips typographus* (IT), exhibiting mass attack to combat the tree defense showed higher fungal diversity among the spruce feeding BB in this study. It is worth to mention here that some fungi such as *Endocondiophora polonica*, *Grosmannia penicillate*, and *Grosmannia europhioides* associated with the aggressive beetles (e.g., *Ips typographus*) often utilize the tree defensive toxic compounds as carbon source and thus reduce their concentration in addition to lessen the competition between symbiotic partners for available carbohydrate like glucose (Zhou et al., 2016; Zhao et al., 2019). Hence, the fungal association may have a dual benefit for bark beetles (BB). However, such assumptions need further experimental corroborations.

In addition to tree colonization behavior, other factors such as sampling site, microenvironment of the habitat, nutrient availability and defensive host compounds influence the gut mycobiome communities in BB. Despite all these variabilities, our data suggest the presence of core fungal communities constituting of 19 genera that were conserved among all five *Ips* bark beetles (Figure 4). The majority of these core fungal genera belong to the phylum Ascomycota. Interestingly, some of the Ascomycota associates (*Grosmannia*, *Ophiostoma*, *Endocondiophora, Ceratocystis*, and *Leptographium*) in the BB (e.g., *Dendroctonus ponderosae*, *Ips typographus*) were reported to endure host tree defense mechanism and utilize these defensive secondary metabolites as carbon sources (Hammerbacher et al., 2013; Cale et al., 2016; Wadke et al., 2016; Zhou et al., 2016; Davis et al., 2018). The detoxification of defensive plant compounds and the ability to exploit these metabolites as carbon sources by the symbiotic fungi benefits the BB and boosts their fitness. Our study revealed the presence of ophiostomatoid fungi *Ophiostoma, Ceratocystis* belonging to Ophiostomataceae and Ceratocystidaceae family, as the core gut members in the BB from Scolytinae subfamily. Several studies have already documented ophiostomatoid fungi (e.g., *Grosmannia penicillate*, *Ophiostoma bicolor*, *Ophiostoma piceae*, *Ceratocystis polonica*, *Leptographium chlamydatum*) to be frequently associated with *I. typographus* (Viiri, 1997; Persson et al., 2009; Linnakoski et al., 2010). These ophiostomatoid fungi, commonly referred to as blue staining fungi, are serious tree pathogens (Wingfield et al., 1993; Kirisits, 2004), and their association with the BB has been formed over a long period (Hartig, 1878). The fungal spores of ophiostomatoid fungi are typically dispersed by BB (Masuya et al., 2009). As the BB invade the coniferous trees and the fungi get transported to the phloem and the bark where they function in nitrogen acquisition and maintains the nitrogen balance that is critical for beetle development (Ayres et al., 2000). Similarly, *D. brevicomis* beetle surviving on the outer bark largely depend on the two mutualistic fungi (*Ceratocystiopsis brevicomi and Entomocorticium* sp.) for maintaining the nitrogen and phosphorous ratios in their diet (Six and Elser, 2019). Furthermore, *Ceratocystis* sp. also contributes to the detoxification of phenolic compounds released as tree defensive response against beetle or fungal attack (Hammerbacher et al., 2013). Such association between the ophiostomatoid fungi (*Ophiostoma ulmi*) and the *Scolytus* spp. BB has been previously reported to kill numerous elm trees in Europe and North America (Hubbes, 1999; Santini and Faccoli, 2015). In contrast to *Ophiostoma* sp., the association of *Ceratocystis* sp. with the bark beetles are not very specific (Wingfield et al., 2016). The fungi *Ceratocystis* are often vectored by different insects and can infect host plants belonging to different orders (Roux et al., 2007; Lee et al., 2016). It is noteworthy that the presence of such fungal genus (*Ceratocystis*) in the core consortium could presumably pose an additional threat to forests, causing serious tree diseases such as Eucalyptus wilt (caused by *C. fimbriata s.l.*) or *Ceratocystis* wilt (caused by *C. manginecans*) (De Beer et al., 2014).

The additional presence of non-ophiostomatoid fungi belonging to the genera *Fusarium, Penicillium*, *Trichoderma, Beauveria*, and *Cladosporium* in the core communities in BB gut were also observed in this study. Similar to the ophiostomatoid fungi, *Fusarium*, an important plant pathogen, was documented to induce necrotic lesion on the phloem and disturb water conduction, ultimately killing the tree (Solheim, 1988; Yamaoka et al., 2015). Previous studies reported the association of *Fusarium* with *Ips sexdentatus* (SX) and detected their presence in SX galleries (Bezos et al., 2018). Moreover, studies showed mutualistic interaction between *Fusarium* with *Hypothenemus hampei* infesting coffee beans (Morales-Ramos et al., 2000). The occurrence of *Penicillium* and *Trichoderma* was also demonstrated in the previous study done by Krokene and Solheim (1996). It is interesting to note that *Fusarium* and *Beauveria* associated with the bark beetle core gut mycobiome in our study are entomopathogenic (Teetor-Barsch and Roberts, 1983; Zimmermann, 2007). The presence of such entomopathogenic fungus in the BB gut might occur through wounds or by sub-lethal feeding of the fungal spores by BB (Reay et al., 2005). Congruently, the presence of entomopathogenic fungus associated with bark beetles (BB) could eventually serve as a potential candidate for biocontrol strategies against forest pests.

Besides, the filamentous fungi, our results revealed the occurrence of yeasts in the BB gut microbiome. The existence of yeasts symbionts (Suh et al., 2003; Lee et al., 2006) was often attributed to nutrient acquisition, detoxification of defensive plant compounds, and production of volatile organic semiochemicals in BB (Davis et al., 2011). Several yeast genera present within the core mycobiome communities include *Kusarishia, Ogataea, Yamadazyma, Candida, Cyberlindnera*, and *Cryptococcus.* The presence of *Kusarishia* and *Ogataea* was also observed in the mountain pine beetle, *D. ponderosae* (Davis et al., 2011). Interestingly, these yeasts are capable of converting *cis*- and *trans*-verbenol to verbenone, anti-aggregation pheromone (Hunt and Borden, 1990). Similarly, *Kusarishia* and *Candida* enabled such interconversion in spruce feeding *I. typographus* (Leufvén et al., 1984). Besides, some *Candida* species have been reported to produce lipases that hydrolyze the long-chain triacylglycerols and play a significant role in metabolic processes (Suh and Blackwell, 2004). The presence of such fungal species in BB gut might serve a similar function. However, such possibilities demand further experimental corroborations.

Often symbionts interact with each other to fine-tune their co-existence within the host, i.e., *Yamadazyma* and *Candida* identified in the core consortium in the present study has been shown to influence the growth of other fungal symbionts *O. montium* in *D. ponderosae* (Adams et al., 2008). The previous studies suggested that yeast isolated from *I. typographus* outcompetes the fungus *Ophiostoma* in culture. Such influence of yeast on other BB fungal symbionts could compromise the beetle performance as this blue staining fungus may act in parallel to contribute to the death of the beetle-infested trees (Furniss et al., 1990). The yeast *Cyberlindnera*, earlier reported as a common symbiont of *Dendroctonus* beetles (Rivera et al., 2009), detected in our study, can degrade starch and lipids. It can also abide by the secondary metabolites in pine and detoxify them (Briones-Roblero et al., 2017). Additionally, the presence of *Cryptococcus* observed in this study was documented previously in *Ips pini* and *Ips typographus*, having a putative role in the production of semiochemicals (Leufvén and Nehls, 1986).

The persistence of core fungal consortium in the bark beetles (BB) in the present study suggested the involvement of gut mycobiome directly or indirectly in host physiology and performance. Nonetheless, the variation in the gut mycobiome illustrated by the β-diversity may be contributed by the factors such as different feeding habits, environmental impact due to different sampling locations and differences in BB species (Figure 5). The BB belonging to *Ips* and non-*Ips* species showed significant differences in their gut mycobiome. Interestingly, different conifer host feeding also shaped the gut fungal communities where *Ips* bark beetle feeding on spruce (IT and ID) were clustered in one clade similar to pine feeders, IAC and SX. It is worth to mention here that, in the present study, autochthonous mycobiota of the digestive tract of BB cannot be distinguished from the mycobiome that is associated with the diet of beetles. Furthermore, the NMDS analysis revealed that BB gut fungal diversity varied significantly based on their sampling locations and was clustered in three distinct groups. The non- *Ips* species PP collected from Kostelec clustered distantly similar to *Ips acuminatus* (IAC) collected from Libavá. The fungus feeding behavior and the influence of varying sampling location possibly placed apart IAC in a separate cluster than other *Ips* species after NMDS analysis. In contrast, separate clustering of PP may be due to the combined contribution of species and sampling location difference. Although bark beetles (BB) collected from Rouchovany were grouped, there was a distinct separation between different *Ips* species collected from the same site. This observation reflects the feeding behavior of BB on the different coniferous hosts and conserved species-specific mycobiont association within them. However, such conclusions need to be investigated further.

It is interesting to note that each of the BB in the present study represented a set of fungal biomarkers that play a crucial role in their physiology and the performance during and after tree infestation. For instance, the biomarker belonging to the fungal family Ceratocystidaceae was able to detoxify the defensive tree compounds released on a beetle or fungal attack (Hammerbacher et al., 2013). Moreover, the genera *Ceratocytosis* belonging to the family Ceratocystidaceae identified as the core member of the gut mycobiome have a broad tree host range, i.e., this fungus was pathogenic to a wide array of trees (Juzwik et al., 2008; Tsopelas et al., 2017) thereby delivers the BB with the opportunity to infest and survive on different tree hosts. Similarly, the fungal species belonging to Ophiostomataceae family was also considered as a potential biomarker contributing to nutrient acquisition through detoxification of secondary metabolites of host trees and thereby supporting in beetle development (Ayres et al., 2000; Cale et al., 2016; Wadke et al., 2016; Zhou et al., 2016). Other yeast biomarkers such as Pichiaceae and Saccharomycetaceae observed in the BB have been reported to impact the growth of other fungal symbionts in the bark beetles (Adams et al., 2008). Such yeast species were also considered to have a putative role in the biosynthesis of anti-aggregation pheromone in beetles (Hunt and Borden, 1990).

## Conclusion

Taken together, the present study identified and explored the core mycobiome community in five *Ips* bark beetle (BB) species of economic importance. Present findings increase the current understanding of fungal composition and α-diversity in BB (five *Ips* and one non-*Ips*) gut. β-diversity analysis further pinpoints toward the importance of sampling location, host-feeding and species differences as critical contributors for shaping up the BB gut-mycobiont. Further screening the mycobiome within beetle food (phloem), decaying wood and wood from BB galleries may provide higher insight into beetle-fungus interaction. Nevertheless, the present study could be of considerable value and interest in forest entomology, providing insights into the gut-specific core fungal communities and offering a foundation for future follow ups on the functional role that core mycobiome community could have in the gut of *Ips* bark beetles.

## Data Availability Statement

The datasets generated during this study are available under NCBI Bio-project PRJNA632703 (Bio-sample accessions SAMN14917923–SAMN14917958, SRA accession PRJNA632703; https://www.ncbi.nlm.nih.gov/bioproject/?term=PRJNA632703).

## Author Contributions

AC, FS, and AR planned the research. AC and AR conducted the experiments, data analysis, and biological interpretation of results. RM, MA, and JS collected the beetles. MA and JS dissected the beetles. AC, RM, FS, and AR prepared the manuscript. All authors approved the manuscript.

## Conflict of Interest

The authors declare that the research was conducted in the absence of any commercial or financial relationships that could be construed as a potential conflict of interest.

## Footnotes

1 http://ccb.jhu.edu/software/FLASH/

2 http://qiime.org/index.html

3 http://www.drive5.com/usearch/manual/uchime_algo.html

4 https://drive5.com/usearch/manual/chimeras.html

5 http://drive5.com/uparse/

6 http://www.drive5.com/muscle/
