# Supplementary material for: Core Mycobiome and Their Ecological Relevance in the Gut of Five Ips Bark Beetles (Coleoptera: Curculionidae: Scolytinae)
Source: Front Microbiol. 2020 Sep 3;11:568853. doi: 10.3389/fmicb.2020.568853 (PMC7496905; doi:10.3389/fmicb.2020.568853)
Supplement: Supplementary file 9 [file Data_Sheet_1.pdf]

# Core mycobiome and their ecological relevance in the gut of five *Ips* bark beetles (Coleoptera: Curculionidae: Scolytinae)

Authors: Amrita Chakraborty[1], Roman Modlinger[2], Muhammad Zubair Ashraf[2], Jiří Synek[2,] Fredrik Schlyter[2,3], Amit Roy [2*]

# Supplementary Information

**Affiliations**:

[1] EVA 4.0 Unit, Faculty of Forestry and Wood Sciences, Czech University of Life Sciences, Prague, Czechia.

[2] Excellent Team for Mitigation, Faculty of Forestry and Wood Sciences, Czech University of Life Sciences, Prague, Czechia.

[3] Department of Plant Protection Biology, Swedish University of Agricultural Sciences, Alnarp, Sweden

*Corresponding Author: Amit Roy, phone: (+420) 224383565, Email: roy@fld.czu.cz;

## Supplementary Table Legends

**Supplementary Table 1:**

Alpha diversity indices representing the gut fungal community richness and diversity in different bark beetles along with the Good's coverage and number of observed species illustrating the completeness of the sample sequencing.

**Supplementary Table 2:**

ANOSIM and MRPP analyses representing the extent of variation among the bark beetle gut fungal communities. The significant differences between the fungal communities in bark beetle gut are represented by positive R values in ANOSIM analysis. Lower observe-delta value in MRPP analysis indicates less variation in fungal communities within the biological replicates of each beetle, while larger differences between the bark beetles are denoted by higher expected-delta values. The variation among beetles is larger than variation within groups is indicated by a positive A-value in MRPP analysis. Significance value < 0.05 indicates significant differences between the bark beetle gut mycobiome.

**Supplementary Table 3:**

ADONIS analysis based on Bray-Curtis method representing the significant differences between the bark beetles. Df denotes the degree of freedom; SS represents sums of squares of deviations, MS stands for SS/Df. F. Model represents F-test value. The R2 value illustrates the ratio of grouping variance and total variance. Values in parentheses stand for Residual Error. The p-value (Pr) <0.05 determines the significant variation between the bark beetles.

## Supplementary Figure Legends

**Supplementary Figure 1:**

Rarefaction Curves. Different samples are designated by different colours and symbols.

**Supplementary Figure 2:**

The relative abundance of gut fungal communities in spruce (*Picea abies*) feeding bark beetles displayed using GraPhlAn. (A) *Ips typographus* (IT), (B) *Ips duplicatus* (ID) and (C) *Polygraphus poligraphus* (PP). The circle illustrating the different taxonomic level range from inside out and the size of circles resemble the species abundance. Different colours stand for different class. The OTU trees represent the predominance of Sordariomycetes, Saccharomycetes, Eurotiomycetes and Dothideomycetes in the spruce feeding bark beetles. PP showed additional dominance of Agaricomycetes and Leotiomycetes. The high abundance of top 40 species is denoted as solid circles.

**Supplementary Figure 3:**

The GraPhlAn display of the gut fungal abundance of bark beetles. (A) *Ips acuminatus* (IAC) and (B) *Ips sexdentatus* (SX) feeding on pine (*Pinus sylvestris*). (C) *Ips cembrae* (IC) feeding on larch (*Larix decidua*). The circle illustrating the different taxonomic level range from inside out and the size of circles resemble the species abundance. Different colours stand for different class except for IAC where the relative abundance is represented at phylum level. The prevalence of Sordariomycetes, Saccharomycetes, Eurotiomycetes, Dothideomycetes, Agaricomycetes and Leotiomycetes was observed in SX while IC

showed the predominance of Sordariomycetes, Saccharomycetes, Eurotiomycetes Dothideomycetes. The high abundance of top 40 species is denoted as solid circles.

**Supplementary Figure 4:**

(A) The evolutionary tree illustrating the top 100 fungal genera in the bark beetles. Different colours of the branches indicate different phyla. Relative abundance of each fungal genus is displayed outside the circle with different colours denoting their abundance in different beetles. The colours correspond to different bark beetles. (B) The relative abundance of top 20 fungal species represented in the taxonomy tree. Different colours indicate different bark beetles and the size of each sector corresponds to the relative abundance. The first number below the fungal genera denotes the percentage in the whole taxon, while the second number represents the percentage in the particular genus.

**Supplementary Figure 5:**

Ternary plot representing the highly abundant (top 10) fungal genera between three different bark beetles in a time. (A) The dominant fungal genera observed between ID, IT and PP (B) IC, SX, and IAC (C) IC, ID, and SX (D) IC, IT and SX (E) IC, PP and SX. The three vertexes represent three bark beetles. The circles represent dominant species, and the circle size indicates the relative abundance. The circles close to the bark beetles indicates a higher abundance of the species in the particular beetle. *Nakazawaea* is dominant in IC, whereas *Cyberlindera* is highly abundant in IT and ID. *Yamadazyma* is prevalent in ID while *Blastobotrys* and *Talaromyces* are dominant in PP.

**Supplementary Figure 6:**

Flower diagram showing the presence of 37 core OTUs shared among the all six different bark beetles in the present study. [*Ips duplicatus* (ID), *Ips typographus* (IT), *Ips sexdentatus* (SX), *Ips cembrae* (IC), *Polygraphus poligraphus* (PP) and *Ips acuminatus* (IAC)].

**Supplementary Figure 7:**

The heatmap representing the Weighted and Unweighted UniFrac distance matrices for estimating the β-diversity indices between bark beetles. Each grid represents the pairwise dissimilarity coefficient between pairwise samples where the Weighted UniFrac distance displayed above and Unweighted UniFrac distance are denoted in brackets.

**Supplementary Figure 8:**

LEfSe analysis illustrating (A) the histogram of LDA scores showing the occurrence of significantly abundant fungal communities (biomarker) in the gut of six bark beetles. The LDA score at log 10 >4 is set as threshold and the length of each bin, i.e. LDA score represents the extent to which the fungal biomarker differs among the groups. (B) The cladogram representing the gut fungal biomarkers in the six bark beetles. The circles radiating from inside to outside denotes different taxonomic level from phylum to genus. Each circle represents a distinct taxon at the corresponding taxonomic level. The relative abundance of each taxon is proportional to the size of each circle. Fungal biomarkers with a significant difference in their abundances are coloured according to the colour of corresponding bark beetle whereas yellowish-green circles denote the non-significant fungal communities. Red and green nodes denote that these fungal species contribute significantly in the group. Letters above the circles describe the fungal biomarker.

## Supplementary Excel Legends

**Supplementary Excel 1**: Raw and assembled read counts with read length.

**Supplementary Excel 2**: QC statistics and qualified clean read counts.

**Supplementary Excel 3**: The OTU table representing the relative OTU abundance in the bark beetles in groups

**Supplementary Excel 4**: Relative abundance of gut fungal communities at different taxonomic level (phylum level, class level and genus level) present in the bark beetles.

**Supplementary Excel 5**: The common and unique fungal communities comparing spruce feeding bark beetles (IT, ID and PP).

**Supplementary Excel 6**: The common core and unique fungal communities comparing pine feeding bark beetles (IAC and SX).

**Supplementary Excel 7:** The overall core and unique gut mycobiome present in *Ips* bark beetles.

**Supplementary Excel 8:** The overall core and unique gut mycobiome present in all six bark beetles.
