# Supplementary material for: Core Mycobiome and Their Ecological Relevance in the Gut of Five Ips Bark Beetles (Coleoptera: Curculionidae: Scolytinae)
Source: Front Microbiol. 2020 Sep 3;11:568853. doi: 10.3389/fmicb.2020.568853 (PMC7496905; doi:10.3389/fmicb.2020.568853)
Supplement: Supplementary file 10 [file Data_Sheet_2.pdf]

Supplementary Table 1. Alpha diversity indices

Sample	Observed Species[¶]	Good's coverage (%)[¶]	Shannon Index[¶]	Simpson's Index[¶]	Chao1[¶]	ACE[¶]
Polygraphus poligraphus (PP)	176.50±6.84	99.90	2.64±0.21	0.67±0.05	210.72±11.63	218.96±13.20
Ips acuminatus (IAC)	100.50±12.30	99.92	2.84±0.13	0.77±0.02	136.38±16.99	139.74±17.68
Ips sexdentatus (SX)	102.67±3.64	99.90	1.46±0.23	0.42±0.08	134.34±6.15	140.90±5.45
Ips cembrae (IC)	105.17±9.74	99.95	1.66±0.38	0.44±0.11	125.63±14.57	132.30±16.86
Ips duplicatus (ID)	86.00±6.23	99.97	1.81±0.28	0.51±0.08	106.19±6.16	109.76±6.76
Ips typographus (IT)	106.67±8.20	99.93	2.67±0.20	0.69±0.05	128.19±9.24	128.75±9.24

[¶] Data representing the mean value ±SEM of six biological replicates for each bark beetle species. SEM denotes the standard error of the mean.

Supplementary Table 2. ANOSIM and MRPP Analysis

Group[ǂ]	ANOSIM		MRPP			
	R-value	P-value	A	observed-delta	expected-delta	Significance
PP-ID	0.95	0.002	0.2523	0.5873	0.7855	0.001
IT-ID	0.3926	0.003	0.07495	0.6074	0.6566	0.024
IT-PP	1	0.001	0.2816	0.5644	0.7856	0.003
IC-ID	0.9815	0.001	0.3309	0.4848	0.7245	0.004
IC-PP	0.9852	0.005	0.3926	0.4418	0.7273	0.001
IC-IT	0.9111	0.001	0.3274	0.4619	0.6868	0.005
IAC-ID	0.6667	0.005	0.2866	0.5565	0.7802	0.002
IAC-PP	0.6778	0.018	0.339	0.5135	0.7769	0.003
IAC-IT	0.6593	0.001	0.2842	0.5336	0.7455	0.004
IAC-IC	0.6667	0.005	0.4315	0.4111	0.7231	0.003
SX-ID	0.9981	0.005	0.3388	0.4905	0.7418	0.003
SX-PP	1	0.004	0.3988	0.4474	0.7443	0.001
SX-IT	0.9722	0.003	0.3329	0.4676	0.7009	0.002
SX-IC	1	0.002	0.4959	0.345	0.6843	0.002
SX-IAC	0.6667	0.005	0.4205	0.4167	0.7191	0.003

[ǂ] *Ips duplicatus* (ID), *Ips typographus* (IT), *Ips sexdentatus* (SX), *Ips cembrae* (IC), *Ips acuminatus* (IAC) and *Polygraphus poligraphus* (PP).

Supplementary Table 3. ADONIS Analysis

Group[ǂ]	Df	SS	MS	F.Model	R2	Pr(>F)
IC-PP	1(10)	2.2284(1.1548)	2.22837(0.11548)	19.297	0.65866(0.34134)	0.001
IC-IAC	1(10)	2.2696(1.2615)	2.26958(0.12615)	17.991	0.64275(0.35725)	0.001
IC-SX	1(10)	2.45632(0.69905)	2.45632(0.06991)	35.138	0.77846(0.22154)	0.001
IC-ID	1(10)	1.8762(1.3983)	1.87619(0.13983)	13.418	0.57298(0.42702)	0.001
IC-IT	1(10)	1.7242(1.2493)	1.72421(0.12493)	13.801	0.57986(0.42014)	0.001
PP-IAC	1(10)	2.1044(1.7475)	2.10440(0.17475)	12.042	0.54633(0.45367)	0.001
PP-SX	1(10)	2.3578(1.1851)	2.35776(0.11851)	19.896	0.6655(0.3345)	0.004
PP-ID	1(10)	1.8060(1.8843)	1.80601(0.18843)	9.5846	0.48939(0.51061)	0.001
PP-IT	1(10)	1.9562(1.7353)	1.95624(0.17353)	11.273	0.52992(0.47008)	0.001
IAC-SX	1(10)	2.1851(1.2918)	2.18510(0.12918)	16.916	0.62847(0.37153)	0.003
IAC-ID	1(10)	1.8093(1.9910)	1.8093(0.1991)	9.0873	0.47609(0.52391)	0.004
IAC-IT	1(10)	1.6379(1.8420)	1.6379(0.1842)	8.8918	0.47067(0.52933)	0.001
SX-ID	1(10)	2.0034(1.4285)	2.00344(0.14285)	14.024	0.58376(0.41624)	0.004
SX-IT	1(10)	1.7805(1.2796)	1.78054(0.12796)	13.915	0.58185(0.41815)	0.001
ID-IT	1(10)	0.56813(1.97880)	0.56813(0.19788)	2.8711	0.22306(0.77694)	0.006

[ǂ] *Ips duplicatus* (ID), *Ips typographus* (IT), *Ips sexdentatus* (SX), *Ips cembrae* (IC), *Ips acuminatus* (IAC) and *Polygraphus poligraphus* (PP).
