# Supplementary figures and images for: Core Mycobiome and Their Ecological Relevance in the Gut of Five Ips Bark Beetles (Coleoptera: Curculionidae: Scolytinae)
Source: Front Microbiol. 2020 Sep 3;11:568853. doi: 10.3389/fmicb.2020.568853 (PMC7496905; doi:10.3389/fmicb.2020.568853)

Supplementary fig. 1

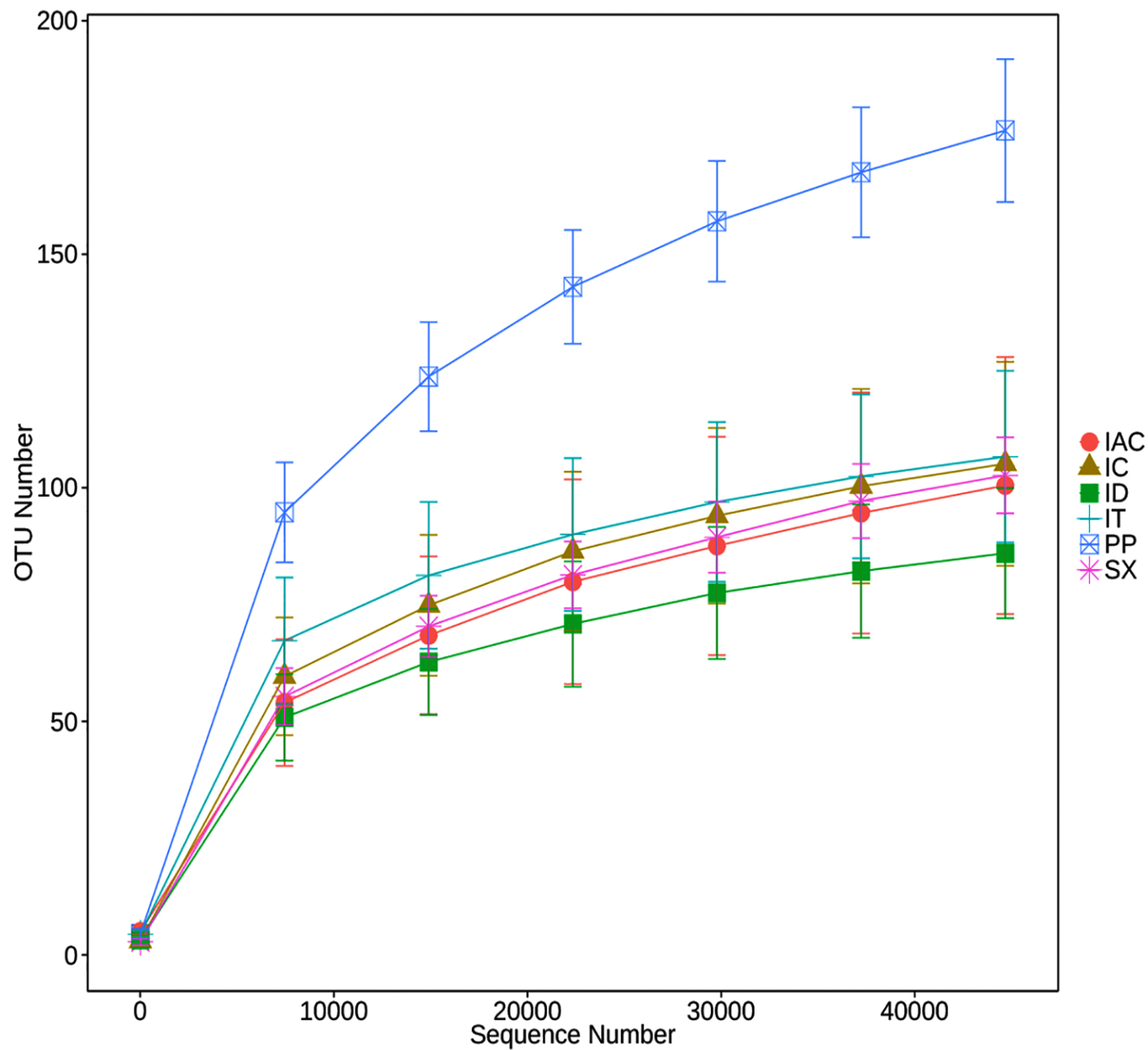

Supplement: Supplementary file 11 [file Data_Sheet_3.PDF]

# Supplementary fig 2

A.

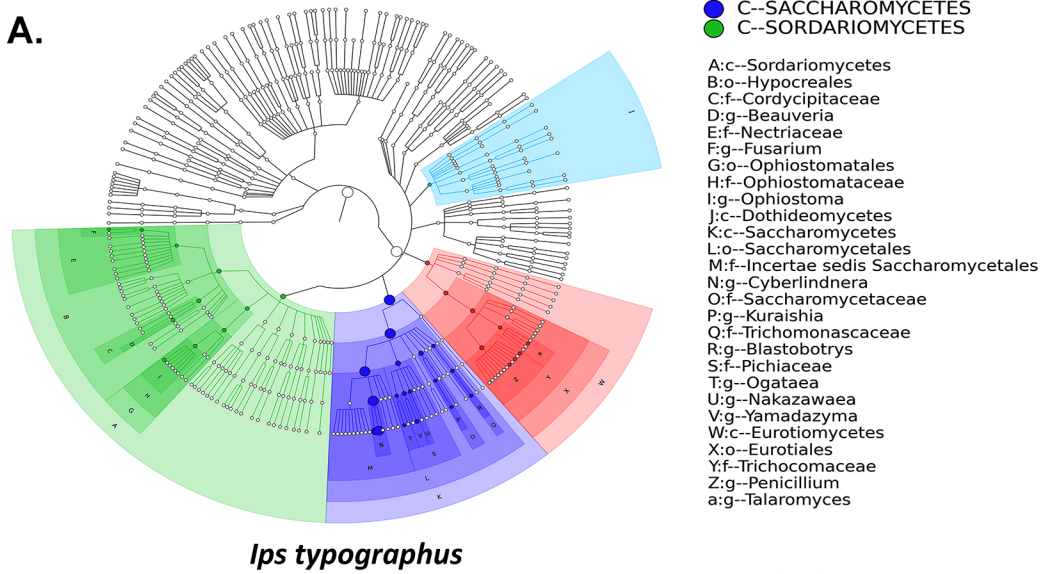

B.

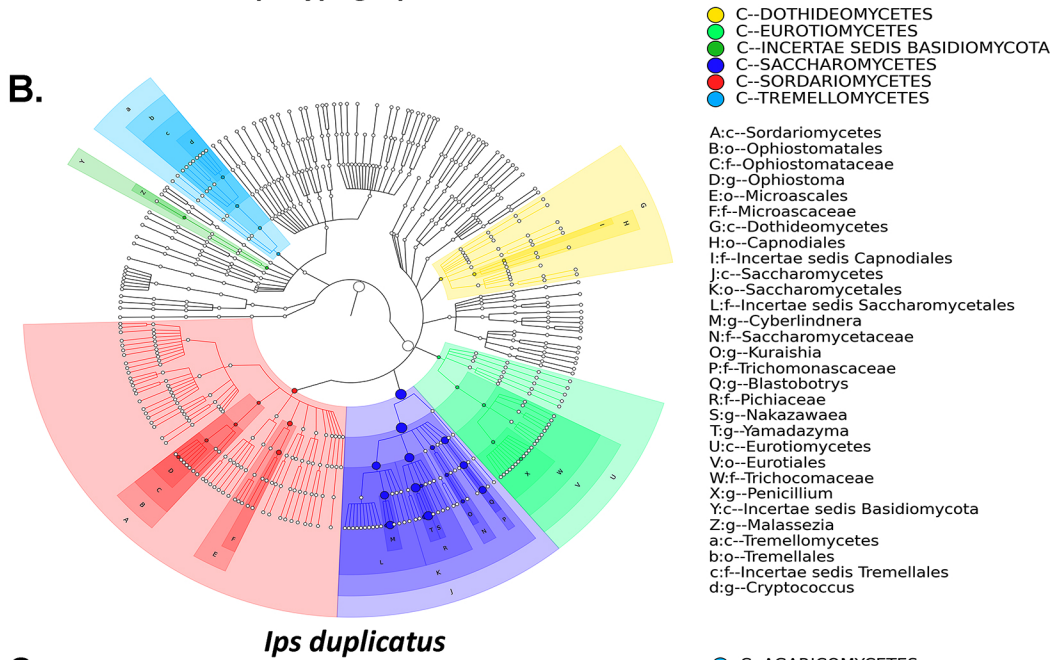

C.

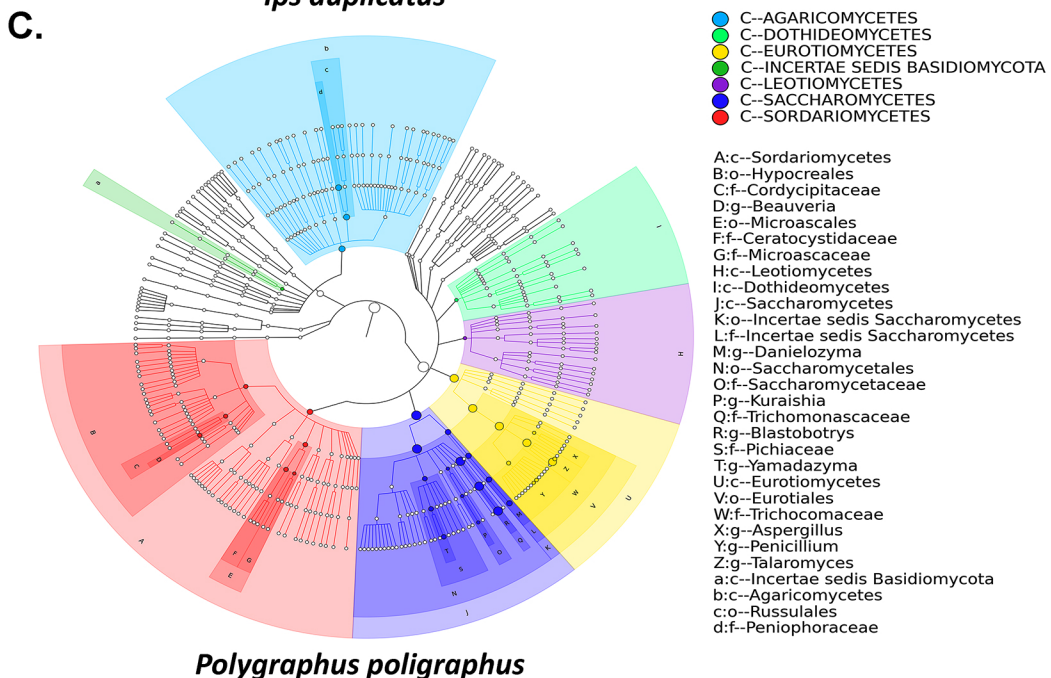

Supplement: Supplementary file 12 [file Data_Sheet_4.PDF]

Supplementary fig. 4

A.

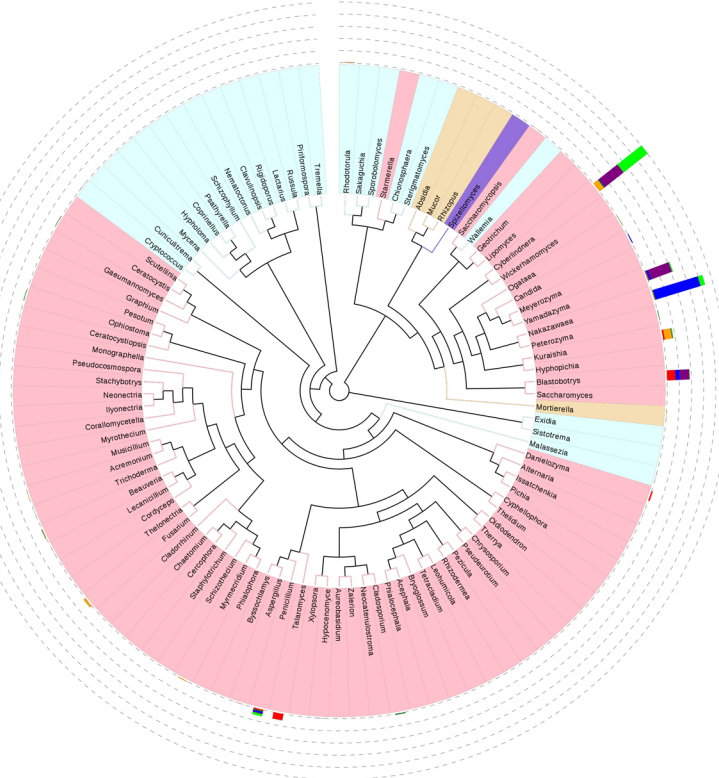

B.

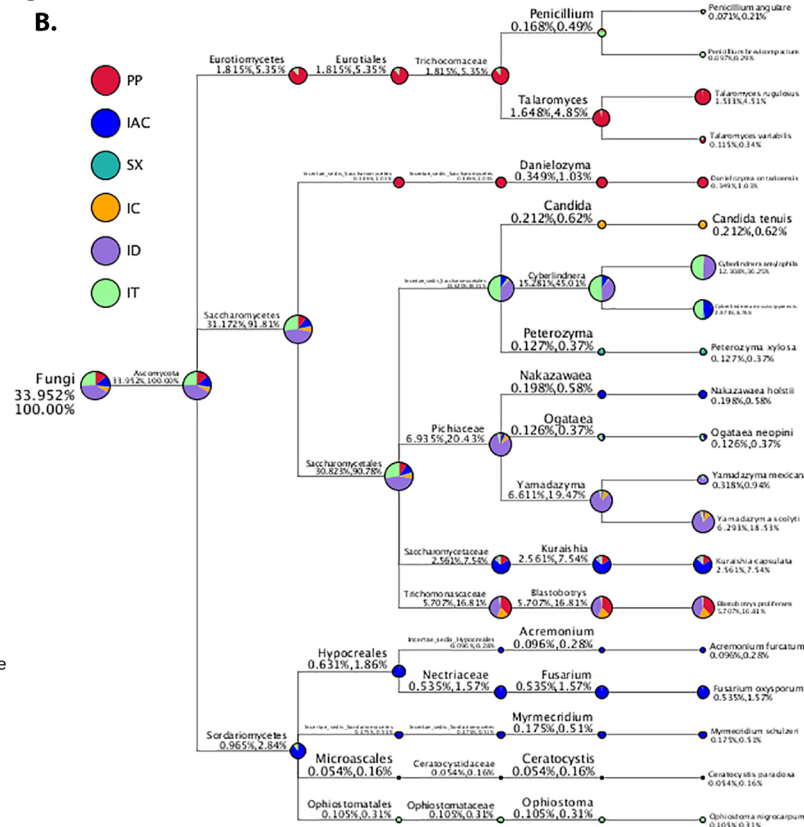

Supplement: Supplementary file 14 [file Data_Sheet_6.PDF]

Supplementary Fig 5

ternaryplot

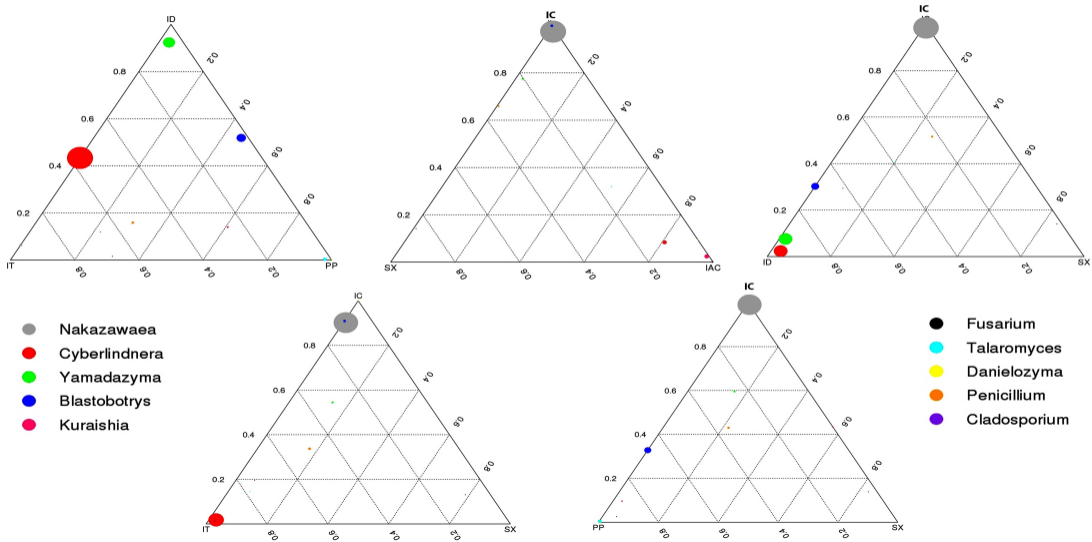

Supplement: Supplementary file 15 [file Data_Sheet_7.PDF]

Supplementary Fig. 6

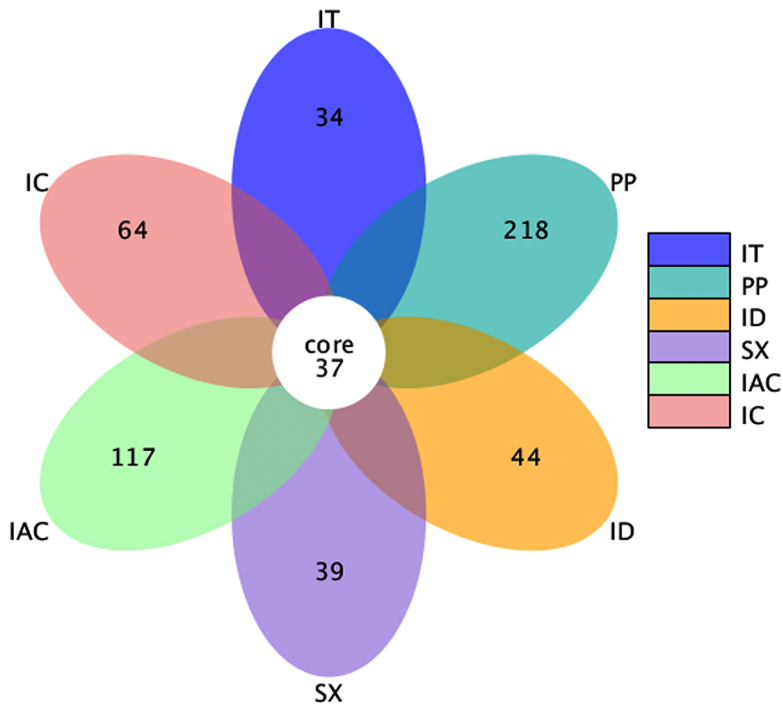

Supplement: Supplementary file 16 [file Data_Sheet_8.PDF]

Supplementary fig. 7

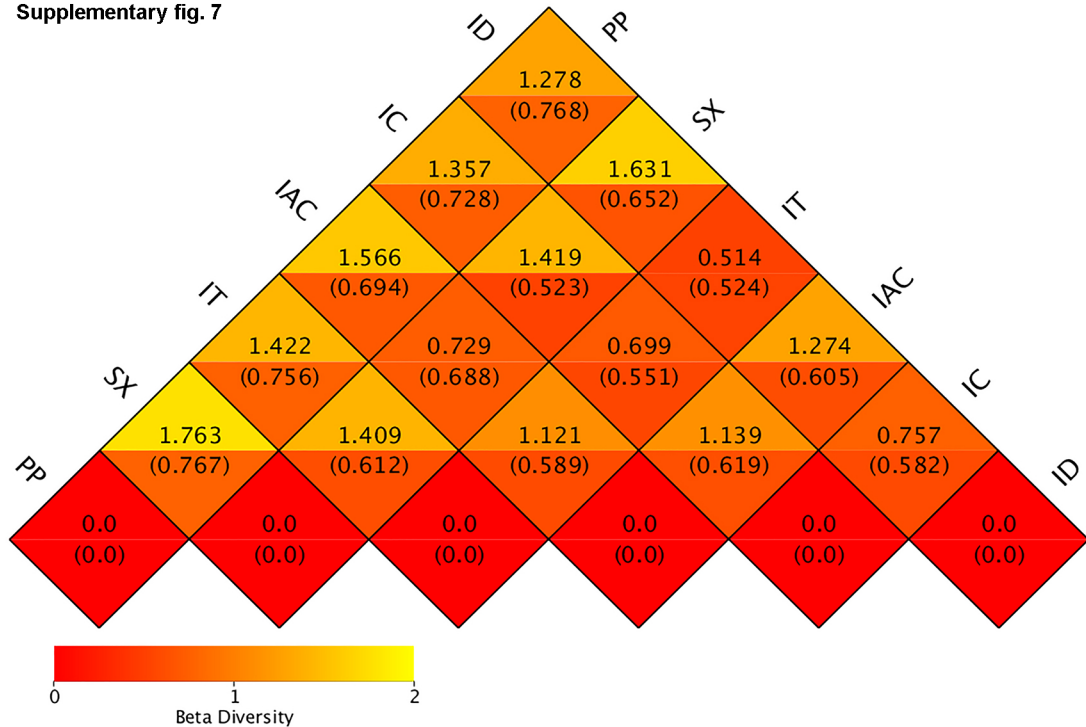

Supplement: Supplementary file 17 [file Data_Sheet_9.PDF]

Supplementary fig. 8

A.

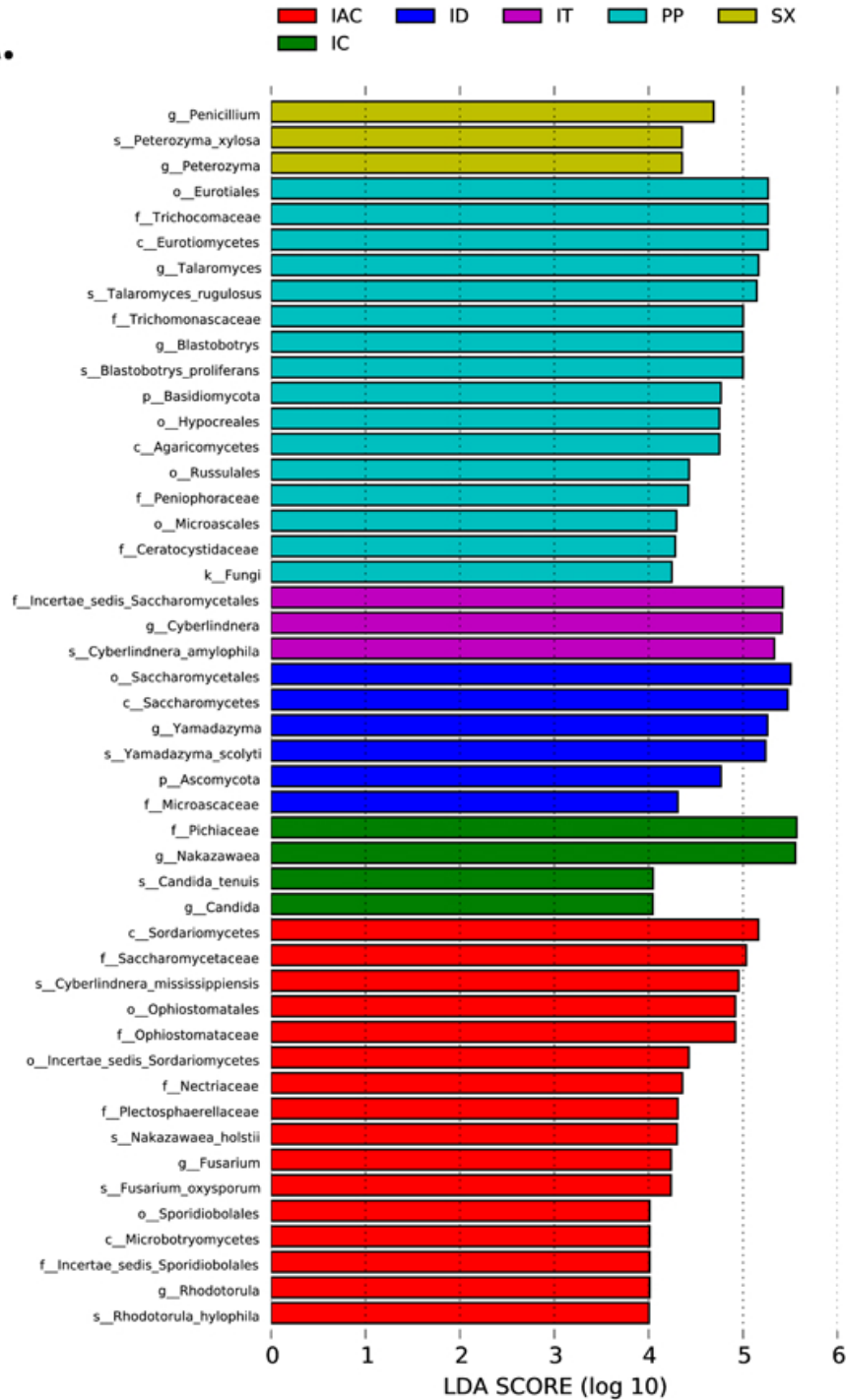

B.

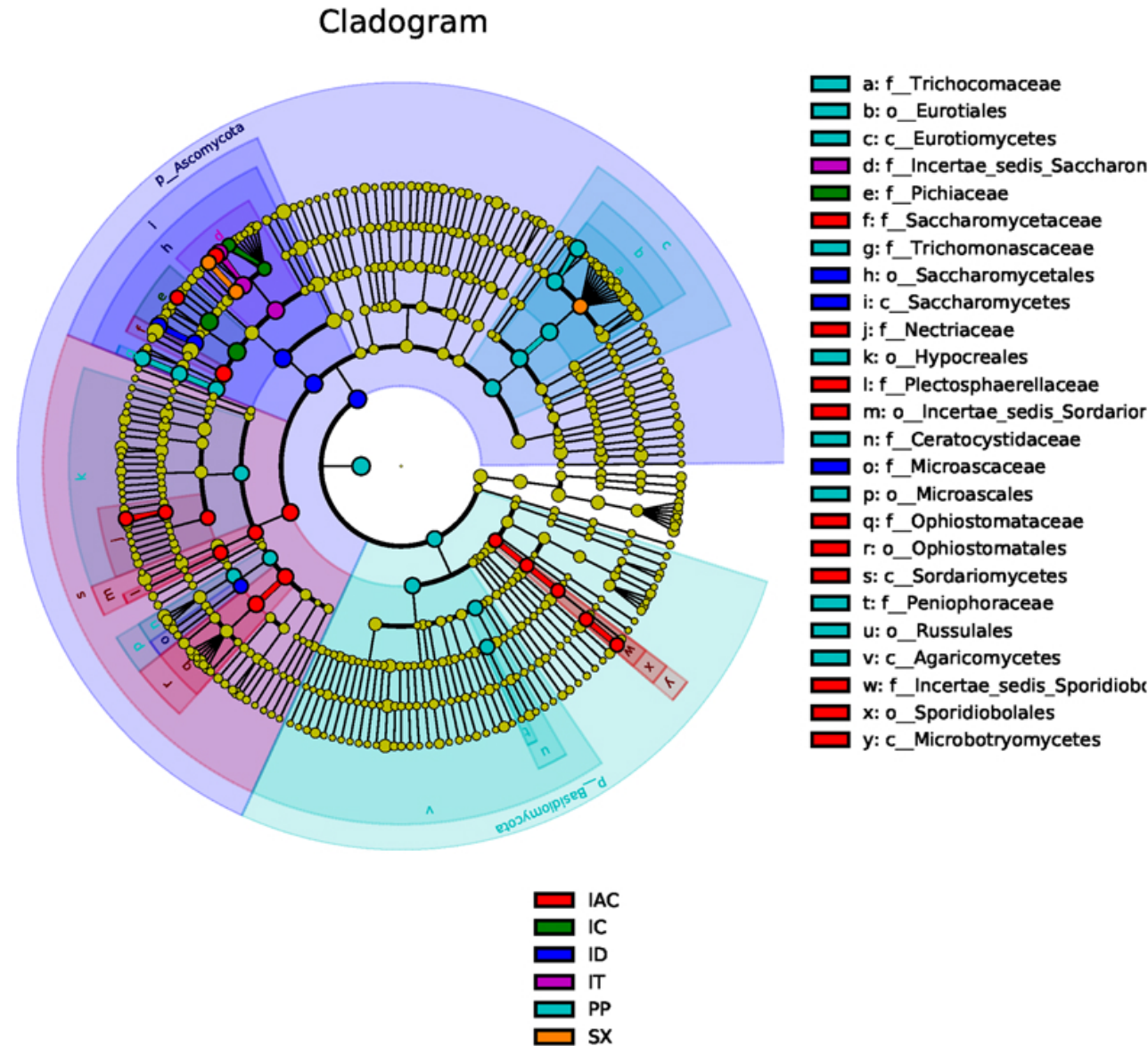

Supplement: Supplementary file 18 [file Data_Sheet_10.PDF]
