# Supplementary material for: Core Mycobiome and Their Ecological Relevance in the Gut of Five Ips Bark Beetles (Coleoptera: Curculionidae: Scolytinae)
Source: Front Microbiol. 2020 Sep 3;11:568853. doi: 10.3389/fmicb.2020.568853 (PMC7496905; doi:10.3389/fmicb.2020.568853)

Supplementary fig. 3

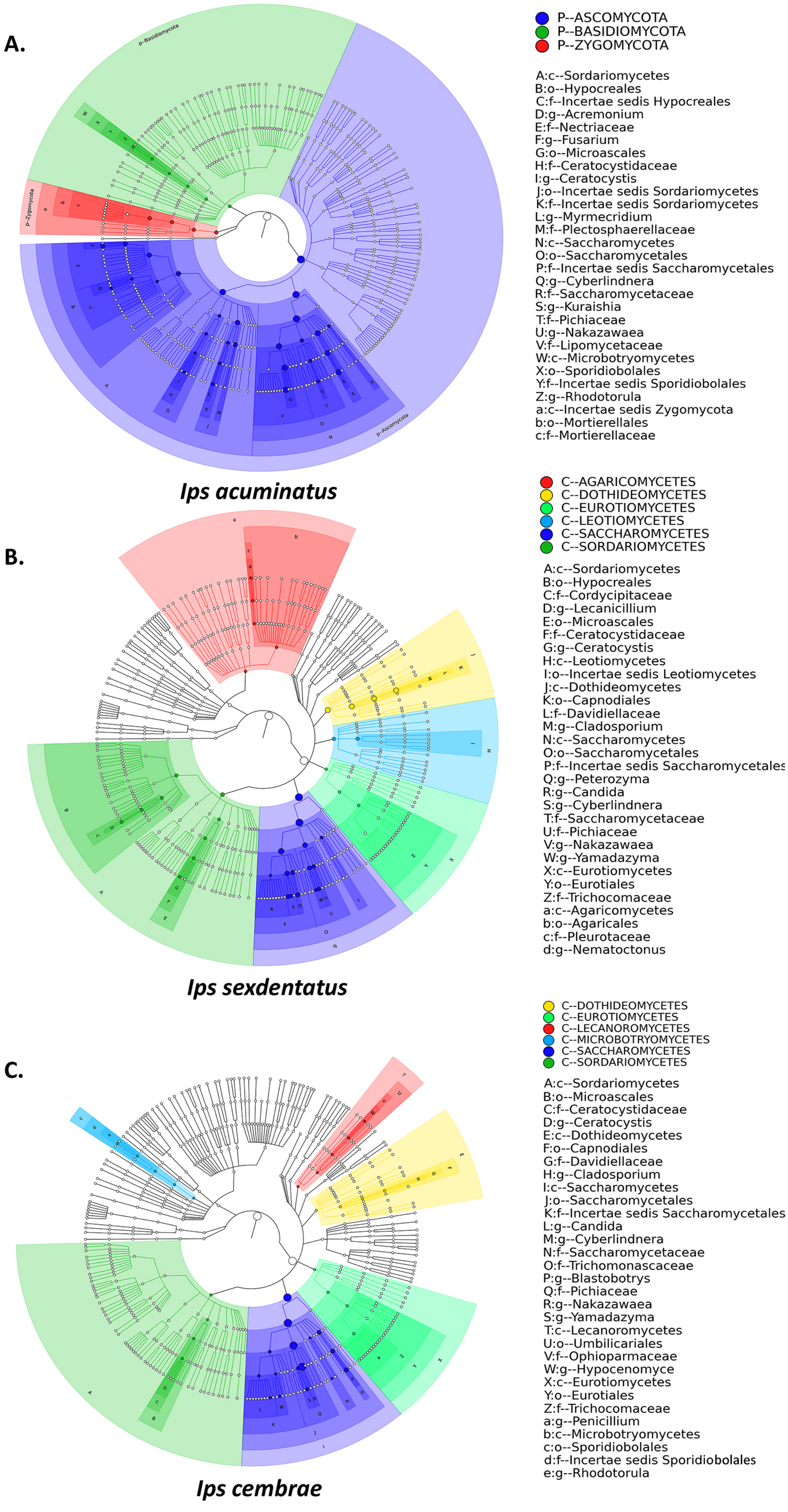

Supplement: Supplementary file 13 [file Data_Sheet_5.PDF]
